# Variations in glacier peak water timing and its influencing factors in High-Mountain Asia

**DOI:** 10.1016/j.fmre.2024.12.006

**Published:** 2024-12-27

**Authors:** Haodong Lyu, Gonghuan Fang, Yaning Chen, Wenting Liang, Zewei Qiu, Yupeng Li, Weili Duan, Zhi Li

**Affiliations:** a State Key Laboratory of Desert and Oasis Ecology, Key Laboratory of Ecological Safety and Sustainable Development in Arid Lands, Xinjiang Institute of Ecology and Geography, Chinese Academy of Sciences, Urumqi 830000, China; b University of Chinese Academy of Sciences, Beijing 100049, China

**Keywords:** Glacier peak water, Influence factor, Future projection, Shared socioeconomic pathway, High-Mountain Asia

## Abstract

High Mountain Asia (HMA) has the largest volume of glaciers outside the polar region and protects approximately 800 million people in the downstream basins against water stress. HMA is facing rapid glacier melting and substantial glacier mass loss, reaching the peak of runoff (‘peak water’) in the following decades. In the present study, the timing of glacier peak water was calculated using a projected glacier runoff dataset forced by twelve General Circulation Models under the Shared Socioeconomic Pathway (SSP) scenarios. The dominant factors that influence the timing of peak water, such as glacial area, elevation, aspect, slope, latitude and debris thickness, were investigated using partial correlation and stratified analysis. Our results demonstrated that, for the entire HMA, glacier peak water was predicted to occur in 2028, 2052, 2057, and 2059 under SSP126, SSP260, SSP370, and SSP585, respectively. For each subregion, Hengduan Shan is projected to reach the earliest peak water in HMA in 2024, 2025, 2021, and 2024 under SSP126, SSP260, SSP370, and SSP585, respectively. In West Tianshan, glacier peak water is projected to occur in 2027, 2036, 2050, and 2050 under SSP126, SSP260, SSP370, and SSP585, respectively. In the West Kunlun, glacier peak water will occur in 2070 under SSP126 and 2080 under SSP245 but will not appear until 2100 under SSP370 and SSP585. Glacier elevation and latitude are highly correlated with peak water timing for the entire HMA, with the partial correlation coefficients being 0.48 and 0.47, respectively (*P* < 0.01). Additionally, earlier glacier peak water normally occurs in glaciers with a small area or steeper slope. Debris can also influence the timing of glacier peak water with a thinner debris cover (< 5 cm) leading to delayed peak water. Our findings indicated that glacier elevation and latitude are highly correlated with glacier peak water timing for the entire HMA.

## Graphical abstract

**Figure:**
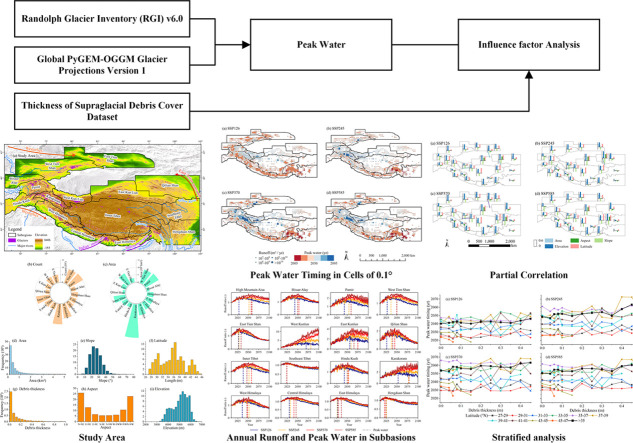

## 1 Introduction

Glaciers are large solid reservoirs and irreplaceable supplies of freshwater, which is vital for human well-being, particularly in extensive cold and arid areas [1]. They act as an important buffer against drought, providing a reliable water source in dry areas where water scarcity intensifies economic and societal vulnerability [2,3]. High-Mountain Asia (HMA) hosts 95,536 glaciers, covering a total area of 97,606 km^2^ [4]. It contains the largest volume of glaciers outside the polar regions, safeguarding approximately 800 million people from water stress [3].

In HMA, the warming rate during 1960–2022 was 0.23  °C per decade [5]. The glacier surface warming rate in the Third Pole is approximately 0.17 ± 0.35 °C per decade [6]. Glacier meltwater in HMA is highly sensitive to climate change and affects the seasonality and amount of water discharge [1,7]. According to the Randolph Glacier Inventory version 6.0 (RGI v6.0) [8], approximately 91.52 % of glaciers have an area smaller than 2 km^2^, collectively covering approximately 32.23 % of the total glacier area in HMA. It has been projected that > 1/3 of the glacierised area and mass will vanish by 2100 under Representative Concentration Pathway (RCP) 2.6 [[9], [10], [11]]. In HMA, glacier mass loss during 2000–2019 was estimated to be 21.1 ± 5.2 Gt/year [12]. For the remainder of the century, glacier mass loss has been projected to be ∼29 % under RCP2.6 and ∼67 % under RCP 8.5 relative to that in 2015, respectively [4].

Alpine glaciers are sensitive to the recent climate warming and are projected to undergo continuous mass reduction throughout the 21st century [4,9,[13], [14], [15], [16], [17]]. As glaciers recede, the annual glacier runoff typically rises until it reaches the peak, often referred to as the ‘glacier peak water’, beyond which it diminishes [18]. After reaching the glacier’s peak water level, the total runoff is expected to decrease, provided that there is no significant increase in precipitation. The resulting water shortage threatens water security, food security, human health, ecosystems, and socioeconomic development in downstream basins and countries [9,10,19]. Previous studies have investigated glacier peak water in HMA based on observed data [9,14,15] or projection models [4,9,18,20]. The timing of glacier peak water varies significantly among subregions [4], and glaciers are projected to experience later peak water under higher emissions [9]. Glacier peak water has already been reached in some regions, including the source regions of the Lancang River and the East Tien Shan [14,15,21]. Future projections indicate glacier peak water in the 2040s, mainly at the source region of the Ili River under all RCP scenarios and at the source of the Yangtze River under RCP6.0 and RCP8.5, as well as in the 2070s, predominantly in the source region of the Tarim River, Indus River, and the northeastern margin of the Tibetan Plateau [4].

Glacier peak water timing is influenced by diverse factors in HMA [4,9]. Previous research has proved that climate scenarios [4,9,[14], [15], [16]], circulation types [4] and glacier area [4,9,18,22] can influence the glacier peak water timing in HMA. It was suggested that glacier peak water always occurs later under the higher emission scenarios in HMA [4,9,[14], [15], [16]]. Glacier peak water varies under varying circulation types, with most basins fed by monsoons predicted to reach their peak water before 2050. Basins dominated by westerlies, including the Indus, are predicted to reach their peak water after 2050 [4]. As for the glacier area, Huss and Hock suggested that basins with higher glacier-covering fractions and larger glaciers tend to experience later peak water [18]. Earlier peak water has been projected for smaller individual glaciers in HMA, especially for those smaller than 2 km^2^ [4,9,18,22]. Furthermore, glacier attributes, including glacier area, elevation, aspect, slope, and thickness and area of glacial surface debris, affect the glacial runoff, which influences the peak water thereafter [4,9,16,23]. Previous research has largely concentrated on the impact of climate change on glacier melt runoff [4,9,[14], [15], [16]]. In recent years, numerous studies have demonstrated that circulation patterns [4] and glacier area [4,9,18,22] also play significant roles in determining the timing of glacier peak water in High Mountain Asia (HMA). However, there has been insufficient attention given to identifying the dominant glacier attribute that influences peak water under a comprehensive analytical framework. This study aims to address this critical research gap by examining the various influences of glacier attributes on peak water timing at the individual glacier scale, thereby enhancing our understanding of the factors that affect the timing of glacier peak water. We investigated the glacier peak water timing of 95,536 individual glaciers in HMA. The analysis utilised glacier runoff projections from the Python Glacier Evolution Model (PyGEM) and Open Global Glacier Model (OGGM), driven by 12 General Circulation Models (GCMs) under four shared socioeconomic pathway (SSP) scenarios (SSP126, SSP260, SSP370, and SSP585). In order to explore the factors, we used partial correction to analyse the influence of area, elevation, latitude, aspect, and slope and stratified analysis to explore the influence of surface debris thickness. We also elaborate on the mechanisms of how these factors influence the glacier peak water timing.

## 2 Study area and data

### 2.1 Study area

**Fig. 1:**
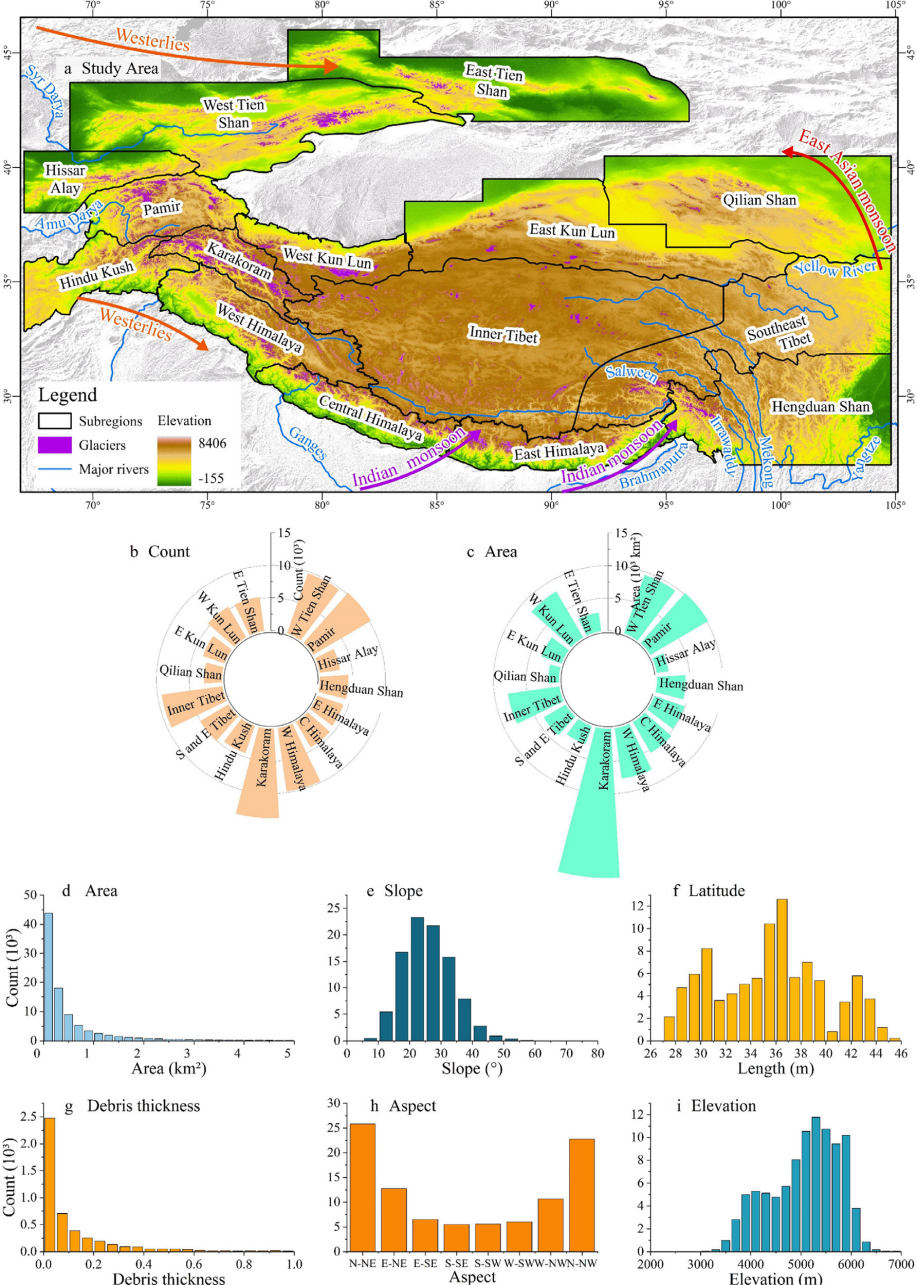
**Location map of High-Mountain Asia (HMA) and attributes of 95,336 glaciers in HMA.** (a) Spatial distribution of glaciers with 15 subregions and dominant circulatory system. (b and c) Distribution of glacier area and glacier number for the 15 subregions. (d–g) Glacier attributes, including (d) area, (e) slope, (f) length, (g) debris thickness, (h) aspect, and (i) elevation. All maps in this study are generated based on the standard map GS(2022)1873.

HMA covers an area of approximately 5 × 10^6^ km^2^ (65°–105°E, 26°–46°N) [4,6,13,24]. The region includes 95,336 glaciers covering 97,606 km^2^ [8], including 2nd-order subregions 13, 14, and 15 in the Randolph Glacier Inventory (version 6.0) (RGI v6.0) [25]. To account for climatic heterogeneity, we divided HMA into 15 subregions: Hissar Alay, Pamir, West Tien Shan, East Tien Shan, West Kunlun, East Kunlun, Qilian Shan, Inner Tibet, Southeast Tibet, Hindu Kush, Karakoram, West Himalaya, Central Himalaya, East Himalaya, and Hengduan Shan. The climate change trends of each subregion are similar. Subregions south of HMA, i.e., the Central and East Himalayas, are controlled by the Indian monsoon. The southeastern margin regions, namely Qilian Shan, Southeast Tibet, and Hengduan Shan are affected by the East Asian monsoon [6,8,26,27] (Fig. 1).

### 2.2 Glacier inventory data

RGI v6.0 [8] records global glacier outlines and attributes of 95,336 glaciers in HMA with the glacier attributes of area, minimum and maximum elevation, median elevation, aspect, and mean slope of the glacier surface. The aspect of each glacier was calculated as the aspect south of the glacier. Spatial distribution, area, and count statistics of the 95,336 glaciers in HMA are shown in (Fig. 1a–c). The glacier attributes of area, slope, length, debris thickness, aspect, and elevation are shown in (Fig. 1d–i).

### 2.3 Future glacier runoff data

Future glacier runoff data were collected from Global PyGEM-OGGM Glacier Projections Version 1 [28]. Runoff projection was generated from a hybrid glacier evolution model combining the mass balance module of the PyGEM and the ice dynamics module of the OGGM. The monthly runoff for each glacier in HMA during 2000–2100 was projected using twelve GCMs: BCC—CSM2-MR, CESM2, CESM2-WACCM, EC-Earth3, EC-Earth3-Veg, FGOALS-f3-L, GFDL-ESM4, INM-CM4–8, INM-CM5–0, MPI-ESM1–2-HR, MRI-ESM2–0, and NorESM2-MM. We adopted the 12 climate models because they have been evaluated and shown to perform well in precipitation and surface temperatures in High Mountain Asia (HMA) [29,30]. Runoff was projected under the four SSPs and averaged to obtain a robust future glacier runoff [31].

### 2.4 Thickness of supraglacial debris cover data

Supraglacial debris thickness data were obtained from a dataset generated by McCarthy et al.[32]. The dataset includes mean thickness measurements of glaciers and debris. The spatial resolution is 25 m based on a smoothed Digital Elevation Model segmented with multiple tributaries. The dataset encompasses 4,689 glaciers with areas larger than 2 km^2^ under quality control in HMA. The fraction of the debris-covered area of the 4,689 glaciers in HMA accounts for 36.31 % of the glacier area.

## 3 Methodology

### 3.1 Timing of glacier peak water

Peak water marks the turning point when annual glacier runoff shifts from increasing to decreasing during glacier retreat [9,18]. We used 11-year moving averages of runoff time-series data to determine the time of maximum runoff. The timing of maximum glacier runoff at the turning point was determined to be the timing of the peak water of each glacier. Additionally, we validated our results against long-term measurements from several glaciers that have already reached peak water, such as Urumqi Glacier No 1 (2022–2025), Parlung No 94 Glacier (2018–2019), and Cangla Glacier (2017–2019), which closely aligned with our estimations.

### 3.2 Partial correlation analysis

We used partial correlation analysis to assess the relationship between the timing of glacier peak water of each glacier and associated influencing factors, that is, glacier area, median elevation, aspect, mean slope of the glacier surface, and thickness of debris on the surface. We assumed that the background climate and climate change are analogous for each subregion. Partial correlation is a type of multiple correlation analysis method that characterises the linear correlation between two variables while controlling for the influence of other variables to avoid mutual interference [33]. The glacier attributes of the 95,336 glaciers studied here follow a normal distribution according to the Kolmogorov–Smirnov test (*P* < 0.001)[34]. The partial correlation coefficient was calculated as follows:

(1) $$\begin{array}{c} r_{xy,z}=\frac{r_{xy}-r_{xz}r_{yz}}{\sqrt{\left(1-r_{xz}^{2}\right)\left(1-r_{yz}^{2}\right)}} \end{array}$$

where $r_{xy,z}$ is the partial correlation coefficient between variables x and y, controlling for other variables is represented by z, and $r_{xy}$, $r_{xz}$, and $r_{yz}$ represent the correlation coefficient values. We calculated the partial correlations between glacier peak water timing and each factor while removing the effects of other factors [35]. The p-value was calculated using a *t*-test to determine the statistical significance of the partial correlation results. The results were considered statistically significant if the p-value was < 0.05. Additionally, we also employed Structural Equation Modelling (SEM) methods to analyse the relationship between glacier attributes and the timing of glacier peak water. The findings were consistent with the results using partial correlation analyses (Fig. S15).

### 3.3 Stratified analysis

For the whole HMA, latitude plays an important role in glacier peak water timing because of its large range. We categorised sorted the latitude of glaciers from south to north into 10 categories, with each bin containing 2° of latitude (i.e. 27°–29°N, 29°–31°N, …, 43°–45°N, 45°–47°N). For each latitude bin, the area, debris thickness, and glacier peak water data were further sorted according to latitude. Subsequently, a linear regression between peak water timing and factors of area and debris thickness in each latitude bin was performed to determine the change in peak water timing and explore the role of debris thickness in peak water timing.

## 4 Results

### 4.1 Timing of glacier peak water

**Fig. 2:**
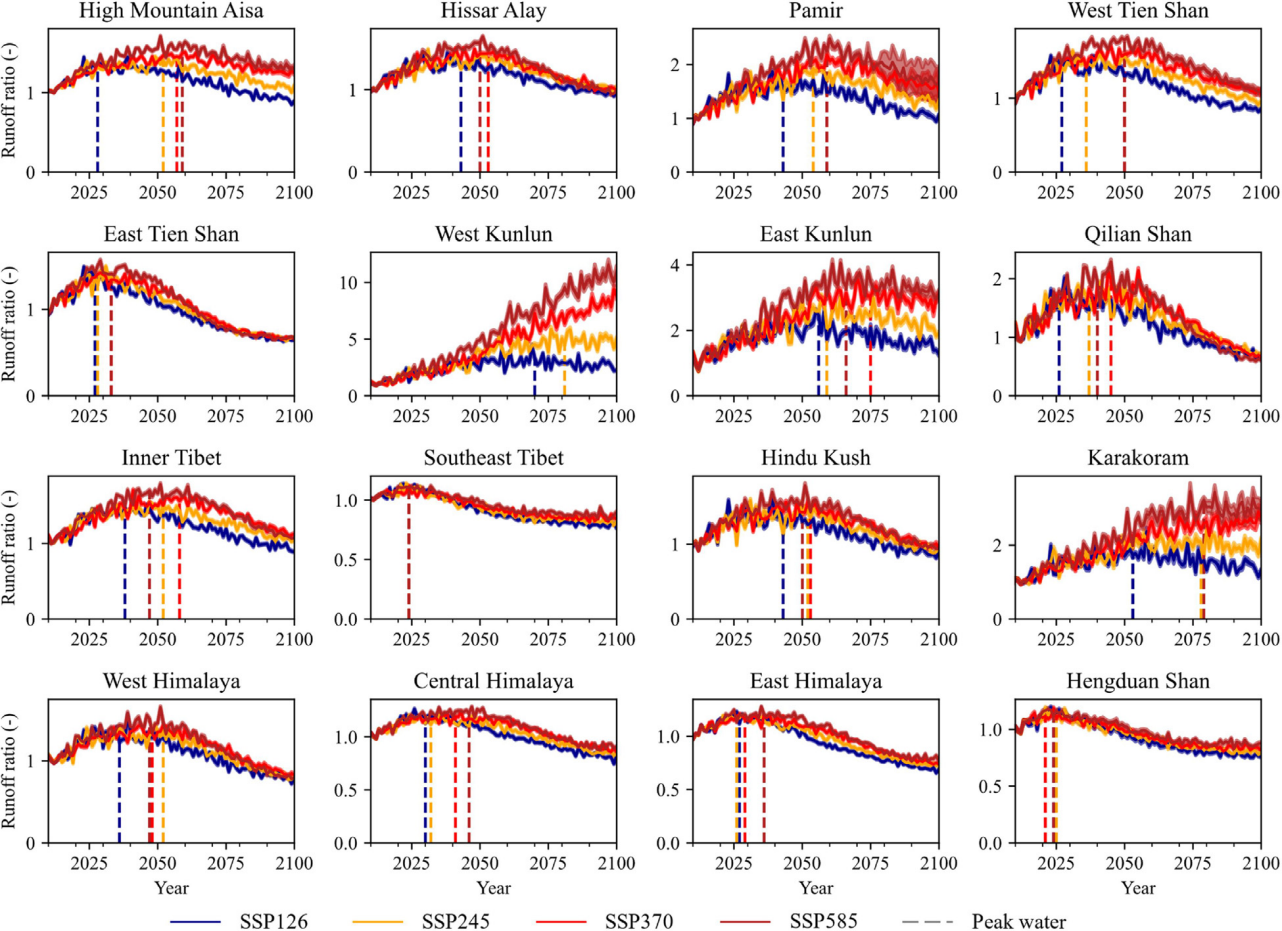
**Variation in glacier runoff ratio during 2015–2100 under four shared socioeconomic pathway (SSP) scenarios: SSP126, SSP245, SSP370, and SSP585.** The runoff ratio was calculated as the ratio of subregional total glacier runoff during 2015–2100 relative to that during 2000–2015. The bold line indicates the ensemble mean, while the shaded area indicates the standard deviation of the 12 simulations. The identified glacier peak water timing is indicated by dashed lines.

For the entire HMA, glacier peak water was predicted to occur in 2028, 2052, 2057, and 2059 under SSP126, SSP260, SSP370, and SSP585, respectively (Fig. 2). The projected peak water varied significantly among different subregions of HMA. Glaciers were predicted to experience their glacier peak water before the end of the 21st century in almost all subregions and SSP scenarios, except for West Kunlun, where glacier peak water was not predicted to appear in the 21st century under SSP370 and SSP585. Specifically, 4 out of 15 HMA subregions (i.e., Central Himalaya, East Himalaya, East Tien Shan, and Hengduan Shan) were predicted to experience glacier peak water in the 2020s under all SSPs. For the eight subregions of Hissar Alay, Pamir, West Tienshan, West Kunlun, East Kunlun, Inner Tibet, Hindu Kush, and Karakoram, peak water was not predicted to appear before the 2050s under SSP585. The uncertainty of the simulation among the 12 climate models increased with time, with the standard deviation ranging from 1.25 × 10^9^ m^3^/year during 2000–2010 to 1.99 × 10^9^ m^3^/year during 2090–2100 for the entire HMA.

**Fig. 3:**
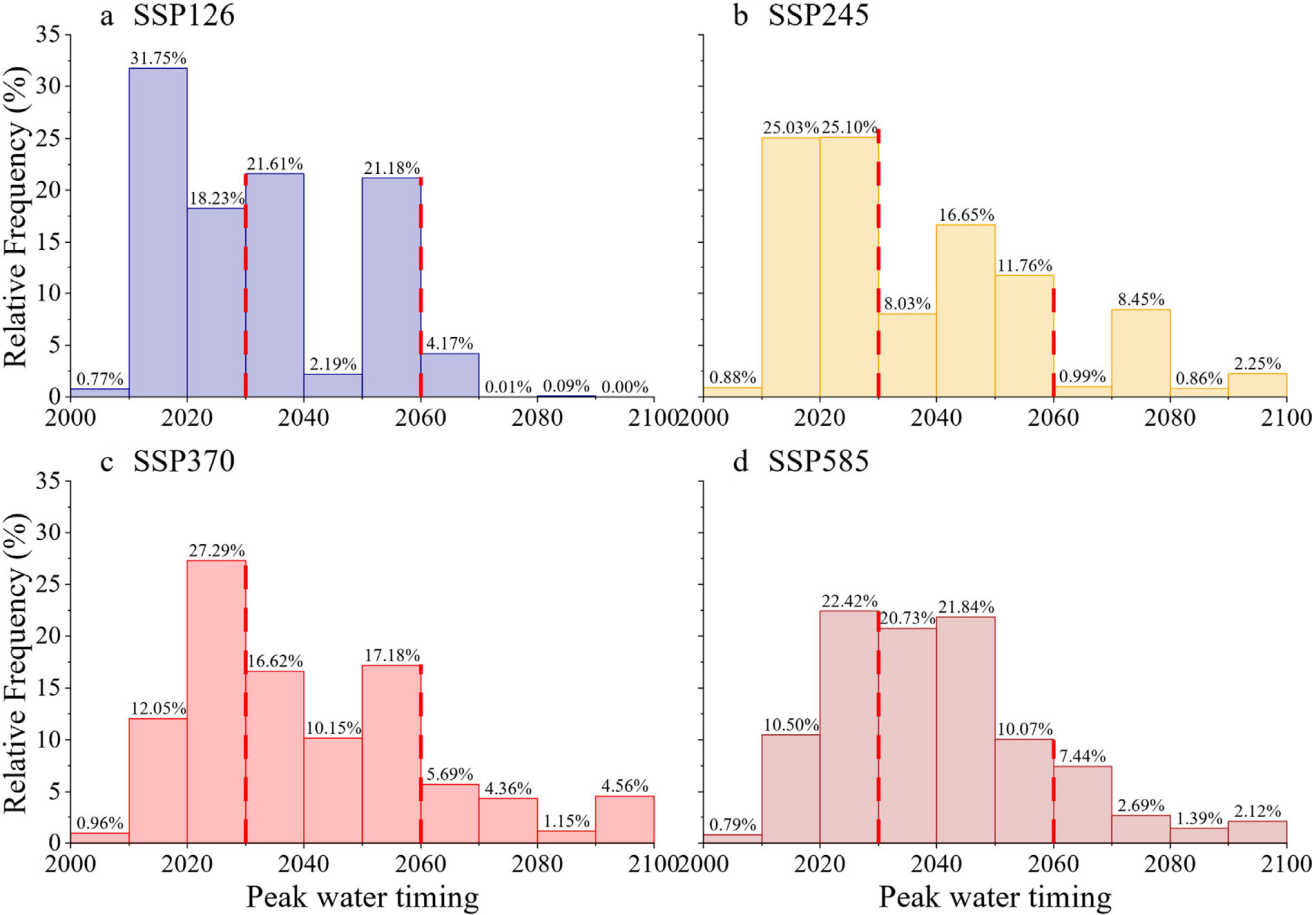
**Glacier peak water distribution for each glacier in High-Mountain Asia (HMA) under four SSP scenarios: (a) SSP126, (b) SSP245, (c) SSP370, and (d) SSP585.** The vertical dashed lines indicate the years 2030 and 2060.

For individual glaciers, approximately 52.05 % (95.76 %), 52.94 % (87.52 %), 40.72 % (84.32 %), and 41.40 % (86.45 %) of the glaciers will experience peak water before 2030 (2060) under SSP126, SSP245, SSP370, and SSP585, respectively. The median and mean timings of glacier peak water were found to be 2028–2034 and 2032–2040, respectively (Fig. 3).

**Fig. 4:**
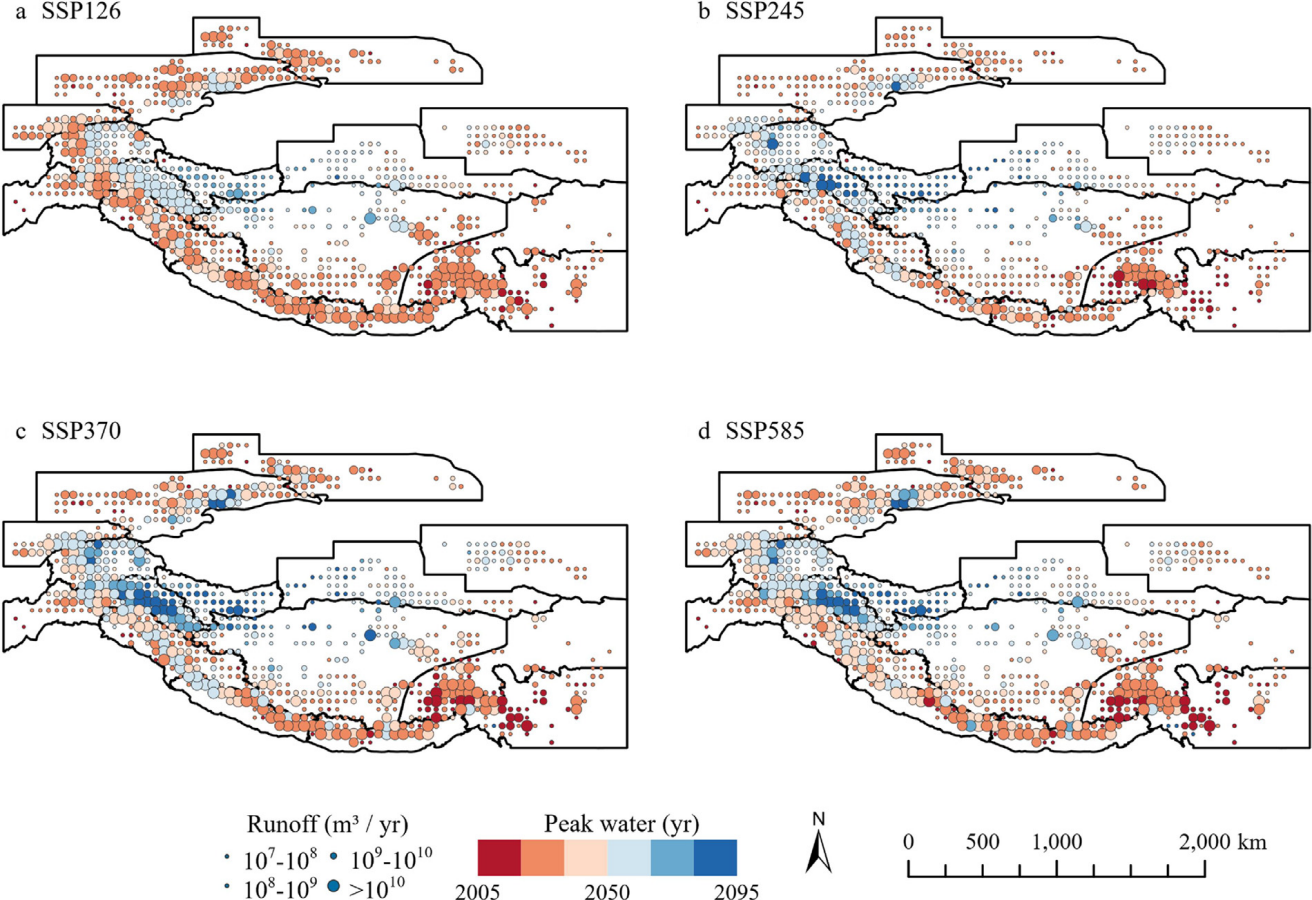
**Spatial distribution of peak water aggregated by 0.5° cells under four SSP scenarios: (a) SSP126, (b) SSP245, (c) SSP370, and (d) SSP585.** The circles represent the ensemble mean of the simulated glacier runoff (m^3^/year) using PyGEM-OGGM forced by multi-General Circulation Model (GCM) outputs.

Fig. 4 shows the glacier peak water timing at a grid scale (0.5° × 0.5°) for clear spatial representation. The spatial variations in peak water timing were similar under the four scenarios, with later peak water observed in higher emission scenarios (SSP370 and SSP585). The earliest grid-averaged peak water was detected in approximately 2005 in Hengduan Shan. The latest peak water was predicted to occur in northwestern Karakoram, southwest Kunlun, western part of East Kunlun, and northern Inner Tibet. Significant variations within subregions were also observed. For instance, in Inner Tibet, the timing of the latest and earliest peak water varied between 2019 and 2095 under the SSP245 scenario (Fig. 2)

**Fig. 5:**
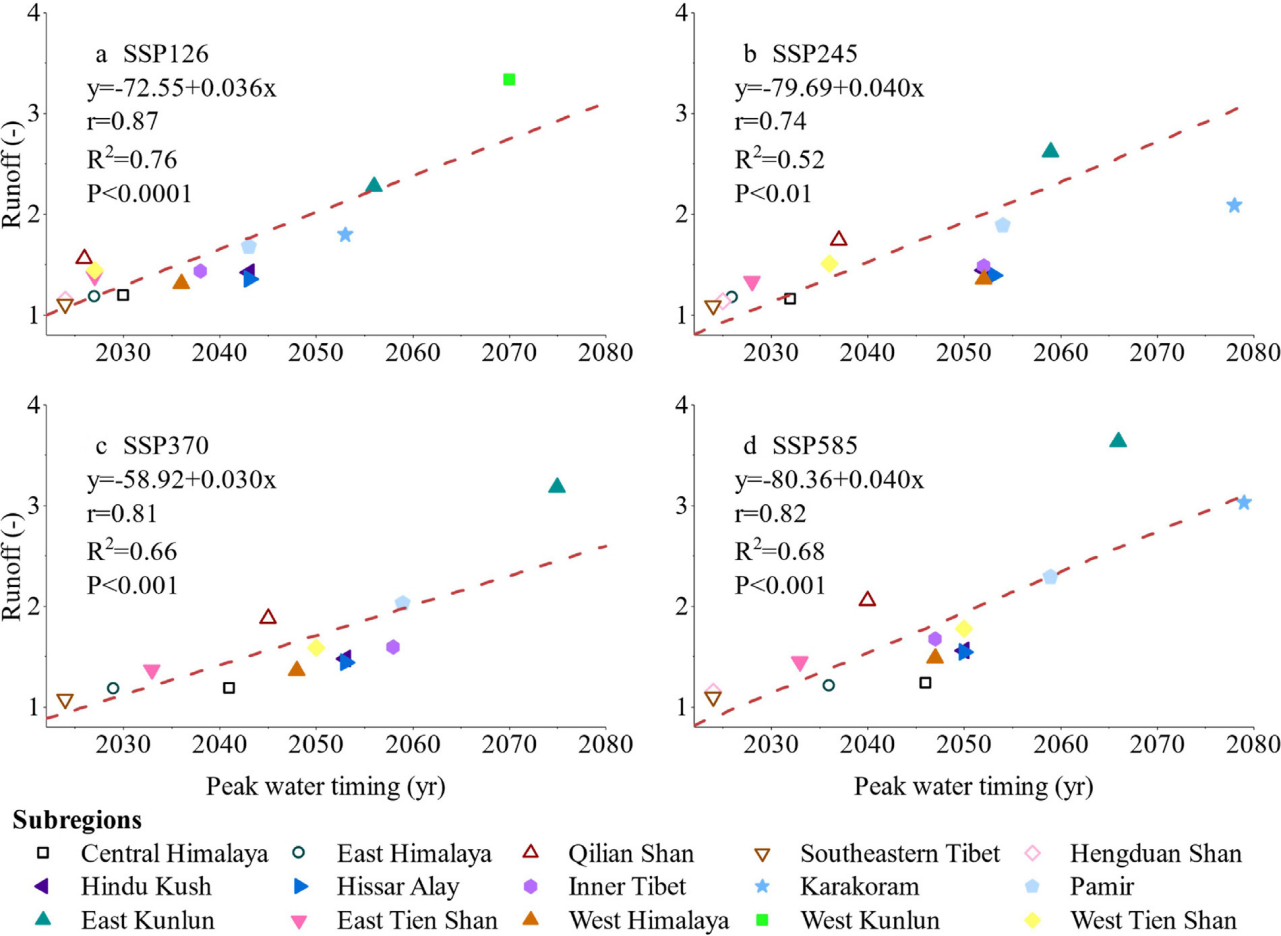
**Glacier peak water timing and the runoff ratio under four shared socioeconomic pathway (SSP) scenarios: SSP126, SSP245, SSP370, and SSP585. The runoff ratio is the mean value of the 11 years centred by peak water year.** The hollow and solid symbols represent subregions controlled by monsoon and westerlies, respectively.

The glacier peak water timing is significantly related to peak water amount, with correlation coefficients of 0.87, 0.74, 0.81, and 0.82 under SSP126, SSP245, SSP370, and SSP585, respectively. The total runoff amount during peak water was 2.91 × 10^11^ m³ under SSP585, which is substantially higher than that under SSP126 (2.34 × 10^11^). The runoff amount and timing of peak water under SSP 370 are comparable to those under SSP585 in most subregions (Fig. 5).

For each subregion in HMA, higher emission scenarios were associated with higher runoff magnitudes, with a correlation coefficient of 0.74–0.87 under the four SSPs. A smaller increase in runoff was observed in monsoon-controlled subregions near peak water (Fig. 5). For Southeast Tibet and Hengduan Shan, which are influenced by monsoons, peak water is predicted to occur in 2024 under all emission scenarios. The peak water during 2024–2025 is expected to be approximately 0.1 times higher than that during the historical period of 2000–2015. In contrast, in East Kunlun, which is governed by westerlies, peak runoff is projected to be 1.15 to 2.56 times greater than that observed during the historical period of 2000–2015 across the four SSPs.

### 4.2 Relationship between glacier attributes and peak water timing

For the entire HMA, the elevation and latitude of glaciers are the most important factors to glacier peak water timing under all the SSP scenarios, with average correlation coefficients of 0.48 (*P* < 0.01) and 0.47 (*P* < 0.01), respectively. Glacier slope and area have a significant correlation with peak water timing, with the correction coefficients being –0.17 and 0.14, respectively. These aspects are negatively correlated with peak water.

**Fig. 6:**
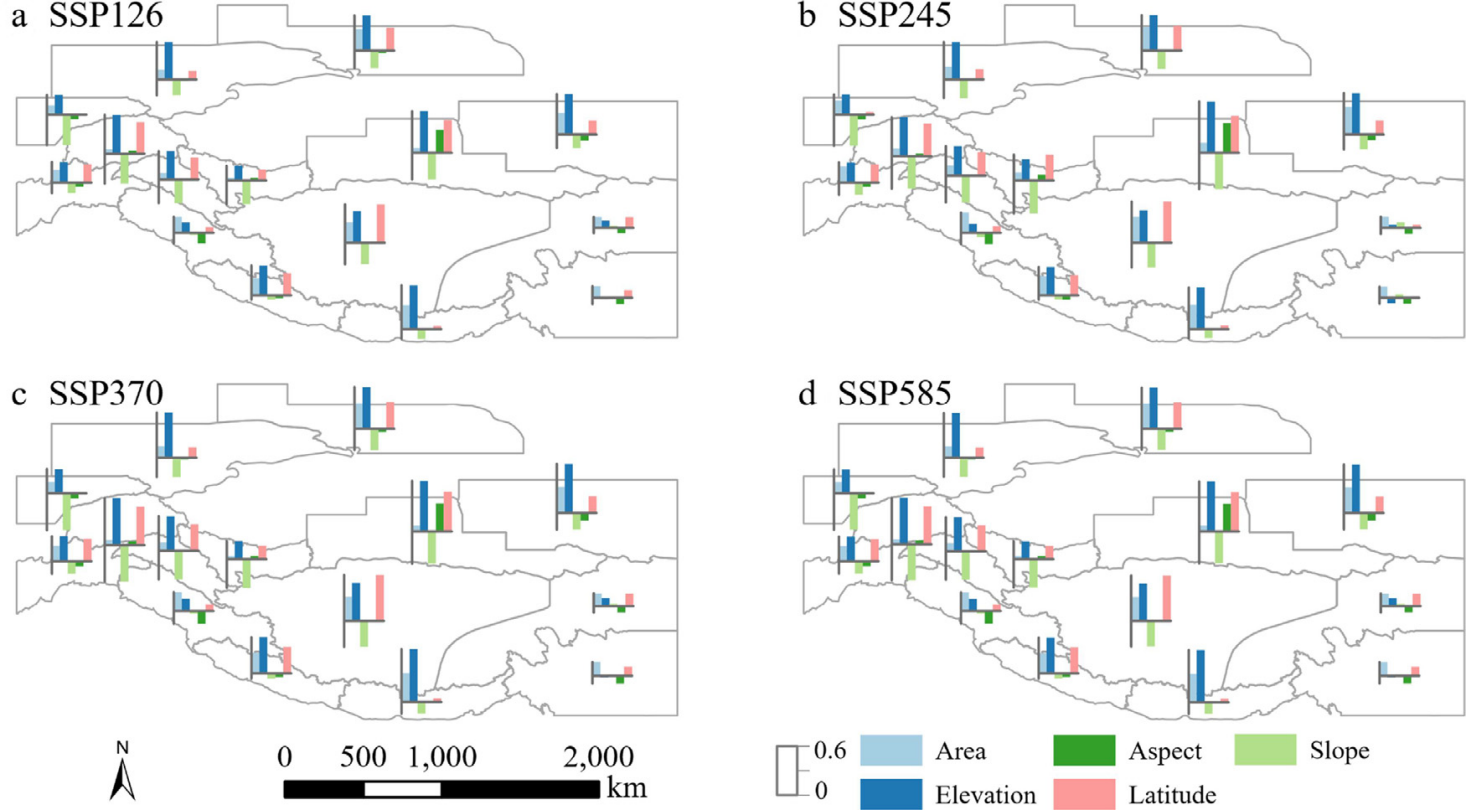
**Partial correlation coefficients of glacier peak water timing and various glacier attributes across each subregion.** The statistics for glacier attributes in each subregion are presented in Fig. 1. Solid bars present a statistically significant correlation between peak water timing and glacier attributes.

For most subregions, accounting for 54.61 % of the area, glacier elevation is the most crucial factor for all emission scenarios, with later glacier peak runoff occurring at higher altitudes (Fig. 6 and S11, S8). In HMA, the subregions with the highest correlation coefficients between elevation and peak water timing are East Kunlun (0.62 under SSP370), Qilian Shan (0.57 under SSP370), East Himalaya (0.49 under SSP370), and Pamir (0.50 under SSP585).

Peak water was mainly predicted to occur during the period of 2020–2040 in glaciers at elevations between 3600 m and 4500 m (Figs. S1, S8). In these subregions, peak water was predicted to occur later in glaciers at higher elevations. In the subregion of Hissar Alay, the partial correlation coefficient between elevation and peak water timing was the largest, indicating a delay of 0.17 years in peak water timing for every 1 km increase in elevation. For Hengduan Shan, peak water was mainly predicted to occur during 2016 and 2028 in glaciers with elevations ranging from 5000 m to 5500 m.

Glacier latitude is also a crucial factor influencing peak water timing, particularly within subregions that exhibit pronounced latitudinal differences among glaciers. These regions are subject to varying rates of climate warming across different latitudes (Fig. 6). Subregions with the highest correlation coefficient between latitude and peak water timing are Inner Tibet (0.48 under SSP245) and East Kunlun (0.46 under SSP370), whereas those with the lowest correlation coefficient were Hissar Alay (−0.01 under SSP126) and Hengduan Shan (−0.12 under SSP585). Glacier latitude is the most important factor affecting peak water in Inner Tibet, with each degree increase in latitude resulting in a delay of 17.09 years in the timing of peak water. For instance, approximately 99.39 % of glaciers situated between 29°N and 31°N are predicted to experience peak water before 2040, whereas approximately 84.17 % of glaciers located between 33°N and 35°N are also predicted to experience peak water before the same year (Figs. S2, S9).

The glacier area is also one of the most important factors affecting peak water timing in HMA, with correlation coefficients ranging from 0.03 to 0.36, for smaller glaciers are more sensitive towards climate warming. Generally, in all subregions under all emission scenarios, earlier peak water was predicted to occur in smaller glaciers (Figs. S3, S10). The correlation coefficients between glacier area and peak water timing are the highest in West Kunlun and West Himalaya, where peak water tended to occur later in glaciers with larger areas and earlier in those with smaller areas. The mean peak water timing of glaciers with areas smaller than 0.2 km^2^ was 2032, 2038, 2042, and 2040 under SSP126, SSP245, SSP370, and SSP585, respectively, being 4 years on average earlier than that of larger glaciers with glacier area larger than 0.2 km^2^ (Fig. S4).

Slope is the factor most negatively related to glacier peak water timing in most subregions of HMA and it is the most important factor affecting peak water in West Kunlun and Southeast Tibet. In Inner Tibet, peak water timing is strongly related to glacier slopes, with an average peak water timing of 2073 for slopes smaller than 15° and 2067 for glacier slopes larger than 15° (Figs. S5, S11). Aspect angle has a negative effect on peak water timing in monsoon-controlling subregions such as Southeast Tibet and Qilian Shan, and a positive effect in some westerly-influenced subregions, including East and West Kunlun (Figs. S6, S12).

The most important factor affecting peak water timing does not differ among the four SSPs in most subregions, except for the subregions of Southeast Tibet, Hengduan Shan, and Central Himalaya. In Southeast Tibet, the most important factor under SSP126, SSP245, and SSP370 is glacier area, which increased from 0.13 under SSP126 to 0.18 under SSP585 (Fig. 6). Slope plays the most important role in peak water timing under SSP585. In the Central Himalayas, elevation plays the most important role under SSP126, SSP245, and SSP585 (0.31–0.35). However, area plays the most important role under SSP585, with a coefficient of 0.24 (Fig. 6). Similarly, in Hengduan Shan, elevation plays the most important role, with a coefficient of −0.15 under SSP585, whereas for the rest of the SSPs, area plays the most important role with a coefficient of 0.13–0.14 for the rest of the SSPs (Figs. S7, S12).

We also employed Structural Equation Modeling (SEM) methods to analyse the relationship between glacier attributes and the timing of glacier peak water. The findings were consistent with the results using partial correlation analyses (Fig. S15).

### 4.3 Effects of debris thickness on glacier peak water timing

**Fig. 7:**
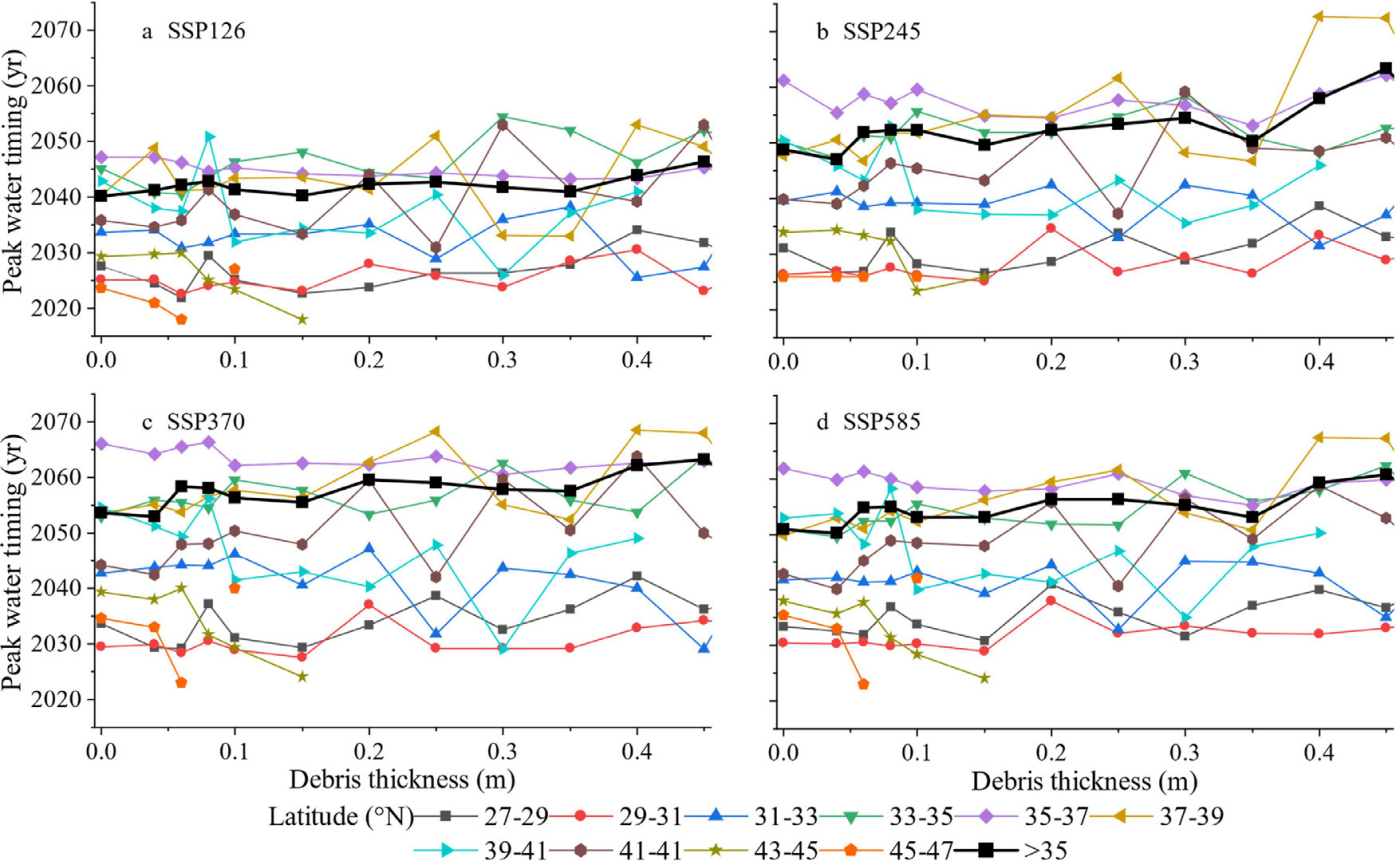
**Detangling the role of debris thickness and latitude in glacier peak water timing.** The subgraphs represent the relationship of peak water timing versus debris thickness, binned by latitude, under the four scenarios of (a) SSP126, (b) SSP245, (c) SSP370, and (d) SSP585 respectively.

In this study, stratified analysis was used to evaluate the effect of debris thickness on the timing of glacial melt peak water. For most latitude ranges, the regression coefficient between peak water timing and debris thickness was negative, except for latitudes 27°–31°N and 33°–35°N. Meanwhile, for latitude 43°–45°N, the effect of debris thickness on glacier peak water timing was strong, with the regression coefficient ranging from −27.69 to 39.19 and a mean debris thickness of 3.66 cm (Figs. 7 and S13, S14). Additionally, peak water timing is negatively related to debris thickness in glaciers with debris thickness of < 5 cm in most latitude ranges (85.22 %). Particularly, at latitudes 27°–37°N and 39°–41°N, the effect is stronger than that of the entire glaciers (Fig. S14).

## 5 Discussion

### 5.1 Projection of glacier peak water

For HMA, we used a glacier runoff projection dataset driven by outputs of 12 GCMs under four SSPs. The results predicted glacier peak water in 2028, 2052, 2057, and 2059 under SSP126, SSP260, SSP370, and SSP585, respectively, for the entire HMA, with the earliest peak water occurring in 2021 in Hengduan Shan. Peak water was not predicted to appear in the 21st century in West Kunlun under SSP370 and SSP585. In each subregion in HMA, certain monsoon-controlled subregions were predicted to experience peak water earlier than others. Peak water was predicted to occur in 2024 in Southeast Tibet and 2021–2025 in Hengduan Shan under all scenarios. This early peak water timing may be attributed to intensified monsoonal precipitation, which is expected to play a larger role in water sources in the future, which are more dominated by monsoonal rain and glacier melt than subregions influenced by westerlies [13]. Increased precipitation will facilitate snow accumulation at high altitudes, which inhibits glacier melting or leads to positive mass balance [27]. Higher summer precipitation caused by monsoons will result in less runoff reduction by the 2100s [4]. In the present study, for the subregions affected by westerlies, peak water was predicted to occur in 2039, 2049, 2055, and 2051 under SSP126, SSP 245, SSP370, and SSP585, respectively. West Kunlun and Karakoram were predicted to experience the latest peak water under SSP370, because increased snowfall partly offsets mass loss, delaying peak water occurrence [4,13]. The estimated timing of peak water is in accordance with that described in previous studies using the OGGM (Zhao Hongyu, 2023 SB), for the years 2037, 2042, 2050, and 2051 under SSP126, SSP245, SSP370, and SSP585, respectively. HMA will experience later peak water than most other regions in the world, including tropical western Canada, low latitudes (Andes), and European Alps, where peak water was observed in the past decades [36,37]. However, peak water in HMA will occur earlier than that in the Copper River Basin (Alaska) and Scandinavia in the years 2070 and ∼2080s, respectively)[37].

For most subregions in HMA, higher emission scenarios typically delay peak water, mainly because higher emission scenarios lead to more glacier runoff and thus delay glacier reduction. Under lower emission scenarios, peak water occurs before small glaciers vanish. However, under higher emission scenarios, it occurs after small glaciers vanish or are unable to offset their mass loss [16,18]. Mass loss is then compensated for by the increased meltwater of large glaciers. Consequently, peak water occurs later under higher emission scenarios than under lower emission scenarios. Peak water was predicted to occur later under SSP370 than under SSP585 in certain subregions, including Karakoram and Inner Tibet, where peak water will occur in 21 and 11 years, respectively. This is consistent with the findings of previous studies on HMA [4,9], although SSP585 refers to the highest additional radiative forcing in the four emission scenarios. This anomaly could be related to the projection uncertainty of variability in small zones of the 12 GCMs simulations in CMIP6.

For each 0.5° cell in the 15 subregions, the spatial heterogeneity of peak water timing in large-range subregions, such as Inner Tibet, reflects the effect of glacier latitude on peak water timing. Additionally, subregions where peak water timing in cells had little variation were predicted to experience an early peak during the following two decades, which is similar to the results of peak water timing evaluations by Rounce et al.[4].

### 5.2 Factors affecting peak water timing

The magnitude and timing of glacier melt peak water are jointly influenced by climatic factors and glacier attributes. Under global warming, summer and spring precipitation will increase at a rate of 46.83 % and 14.00 %, respectively. Temperature, snowfall, and glacier attributes influence variations in glacial melt and accumulation. With increasing glacier melt rate and decreasing glacier area, glaciers reach peak water and then decline, as they release less meltwater from long-term storage (Fig. 1).

**Fig. 8:**
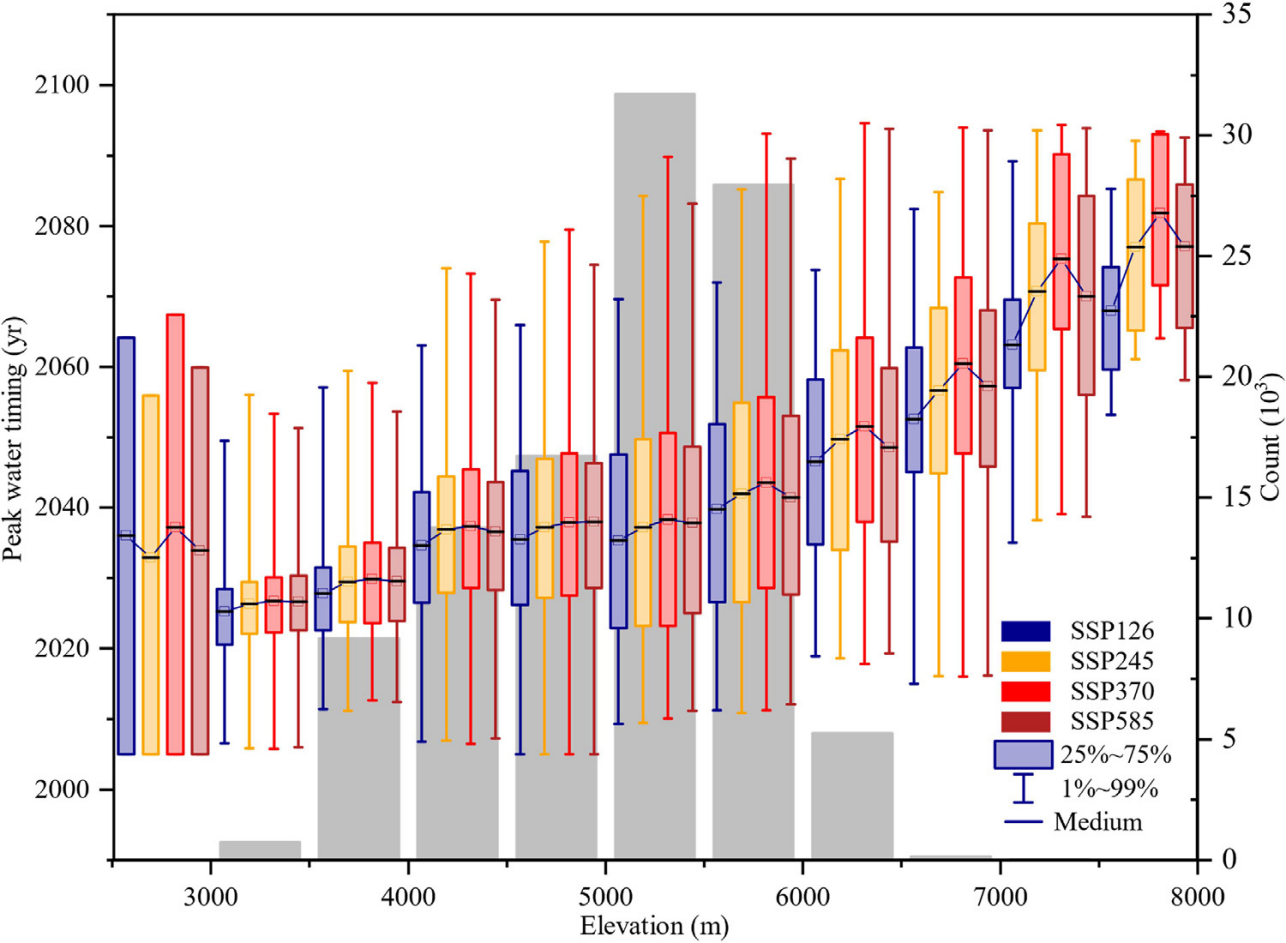
**Accumulation of peak water timing with increasing elevation.** Block and whisker represent 25 %–75 % and 1 %–99 % of the peak water at each elevation, respectively.

Among glacier attributes, elevation was found to be the most relevant factor in most subregions of HMA, owing to its vital role in determining the climatic conditions that affect the rate of glacier melting. In HMA, approximately 68.15 % of glaciers are located at elevations below 5,500 m. For subregions with elevation below 5,500 m, elevation-dependent warming (EDW) was strong and expected to exert an increased influence on the glacier melt rate and decrease the solid precipitation and supply of glacial and snow cover [7,36,38,39]. Below 5,500 m, with an increase in elevation, more glaciers melt, and peak water timing occurs later, especially in subregions with glaciers at a wide range of elevation, such as Qilian Shan, West Tienshan, and East Tienshan. For this reason, high correlation coefficients were observed in subregions where most glaciers are located at altitudes below 5,500 m, that is, West Tien Shan and East Tienshan, whereas lower correlation coefficients were observed in subregions where many glaciers are located at altitudes over 5,500 m, such as West Kunlun and West Himalaya Fig. 8.

The EDW rate varied among different emission scenarios. Under the low-emission scenario of SSP126, there is no discernible gradient in the rate of climate warming with increasing altitude [38]. However, under higher emission scenarios, a notable EDW was observed, with elevation-dependent warming becoming more pronounced as the radiative forcing strengthens [39,40]. Consequently, peak water is more delayed due to EDW under higher emission scenarios than under the lowest emission scenarios of SSP126. However, peak water was predicted to occur later below the altitude of 5,500 m in higher emission scenarios in certain subregions, such as Pamir and Hissar Alay.

In this study, subregions located at higher latitudes were predicted to experience later peak water, which is influenced by other factors besides altitude. These subregions receive less solar heat owing to the reduced angle of solar incidence, resulting in lower-temperature conditions [41]. It is also suggested that the latitude has influenced climate warming, especially in the cold season, and the smaller latitudinal temperature gradient will appear in the future in the HMA [42]. Therefore, peak water will occur later in higher latitudes owing to the higher warming. Thus, latitude is one of the most important factors and is positively correlated with peak water timing. For each subregion in HMA, such as Inner Tibet ranging from 28.95° to 36.09°N, latitude was found to be one of the most important factors. Glaciers in HMA range widely in latitude and are controlled by westerlies. Additionally, the roles of latitude and elevation in climate warming cannot be distinguished. Latitudinal component warming dominates in glaciers at low altitudes, whereas elevational component warming dominates in glaciers at high altitudes in the Tibetan Plateau [42]. Higher coefficients of latitude with peak water timing are mainly recorded in subregions with lower elevations. In East Tien Shan, which has the lowest average elevation of glaciers in HMA, the partial relative coefficient of latitude and peak water is approximately 0.29, which is higher than that of most other subregions.

The glacier area is positively related to peak water timing because small glaciers are more sensitive to climate warming and have higher terminus retreat than those of larger ones [36], which has been shown in HMA [4,16] and other regions such as tropical Andes, Swiss Alps, and western Canada [37]. In China, all the glaciers that have disappeared from glacier basins had a small area, with a mean of only 0.19 km^2^ [23]. For example, 53.03 % of the glaciers in East Tienshan are small glaciers (< 0.2 km^2^). The correlation coefficient between peak water timing and glacier area under the four SSPs was 0.25–0.31. This is significantly higher than that in Karakoram (0.08–0.18), which has the largest average glacier area in HMA (1.66 km^2^). High relative coefficients were detected in subregions with a high proportion of small glaciers, including East Tienshan and Central Himalaya. In the future, earlier peak water can be expected in small glaciers than in larger glaciers at the basin scale [9,17] in HMA [4] and globally [18]. Approximately 47.32 % of glaciers in HMA have areas smaller than 0.2 km^2^, and ca. 16 %, 20 %, and 24 % of them are expected to disappear before 2050 [16] under SSP119, SSP245, and SSP585 respectively, and will reach their peak water in 2030, 2035, 2038, and 2037 (on average) under SSP126, SSP245, SSP370, and SSP585, respectively. This is approximately 3 years earlier than glaciers larger than 0.2 km^2^. This may pose a considerable challenge to water availability in basins dominated by small glaciers, such as Syr Darya and small basins in East Tianshan.

The slope is one of the topographic factors that affect peak water timing by influencing glacial movement and glacier storage. Gentler glacier slopes may experience slower glacier flow, transformation of glacier mass to runoff, slower recharge in the ablation area, and preferential development of glacier ponds [21,43] (Figs. S5, S11). However, steeper slopes can transfer glacier mass more efficiently and adapt more rapidly, thereby reducing glacier mass balance response time to climate warming [23,44]. Meanwhile, steep slopes in combination with the surrounding narrow valleys likely provide a shading effect in lower glaciers, preventing them from absorbing more solar radiation and slowing their melting rates [45]. Consequently, steeper glaciers may experience greater mass loss and earlier peak water, whereas gentler glaciers may experience less mass loss and later peak water. Therefore, glacier surface slope has a negative effect on peak water timing for most subregions in HMA controlled by westerlies. This is particularly important in subregions with a larger slope range and higher altitude, such as East Kunlun and Hissar Alay.

The glacier aspect affects the local-scale solar radiation and air temperature, with south-facing glaciers being more sensitive than north-facing glaciers in the Northern Hemisphere [46]. Glaciers in HMA, similar to other glaciers located in the Northern Hemisphere and heavily affected by strong solar radiation, occur on southern slopes [23]. Therefore, these glaciers tend to melt more rapidly, which can lead to later and higher peak water. For glaciers in West Tienshan, peak water occurs later in the aspects of S–SE and S–SW, with average peak water years of 2034, 2040, 2044, and 2042 under SSP126, SSP245, SSP370, and SSP585, respectively, and earlier for glaciers with aspects of N–NE and N–NW, with average peak water years of 2025, 2029, 2032, and 2031 under SSP126, SSP245, SSP370, and SSP585, respectively (Figs. S6, S12).

The insulation and acceleration effects of debris cover on glacier melting indicate considerable spatial variability among HMA subregions. Generally, thin debris covering less than a few centimetres, typically 0.03–0.05 m, absorbs more solar radiation, which accelerates glacier melting [7,47]. Glacier ablation decreases exponentially with an increasing thickness of thermal insulation. Solar radiation is extremely insulated by debris thicker than a few centimetres [7,48,49].

For the glaciers in HMA, debris thickness has a negative effect on peak water timing for most latitude ranges of glaciers with latitudes greater than 31°N (56.90 % of the glaciers in HMA). As the debris thickness of approximately 60.16 % of glaciers in HMA is greater than 3 cm, it plays an insulating role in absorbing solar radiation, which advances the occurrence of peak water.

For glaciers in the latitude ranges of 33°–35°N, debris has the most accelerating role in absorbing solar radiation due to the proportion of glacial debris thinner than 5 cm, accounting for 61.89 %, larger than most of the other latitude ranges. The thicker the debris, the later the peak water occurs at a latitude range of 33°–35°N (Figs. 7 and S13, S14). It has also been suggested that the acceleration effect debris area accounts for 57 %, which is larger than that of insulation-effect debris of approximately 43 % [47]. The acceleration effect in peak water delay is more important in the glaciers with debris thinner than 5 cm in most latitude ranges (85.22 %) (Fig. S14).

This study had certain limitations. First, it is based on a glacier runoff projection dataset, and calibration with observed data of glaciers in HMA was not carried out, which could have led to some errors in runoff projection despite the small difference in peak water timing between this and previous research. Second, it lacks the calculation and analysis of the shading effect of the surroundings, as this effect is determined by multiple topographic factors, including slope, aspect, and elevation. This could interfere with the correlation between factors. Further research into the glacier attributes influencing peak water timing in HMA requires refining the projection model and considering additional potential factors in future studies.

## 6 Conclusion

In this study, we examined the peak water timing of 95,336 glaciers in the High Mountain Asia (HMA) region and identified potential contributing factors, including glacier area, aspect, slope, latitude, elevation, and subglacial debris thickness, under the SSP126, SSP245, SSP370, and SSP585 scenarios. The main conclusions are summarized as follows: (1) Peak water occurs later under higher emission scenarios of SSP370 and SSP585 than in SSP126 and SSP245, across glacier, grid, and subregional scales. Peak water is projected to be reached in the 2020s for the Central Himalaya, East Himalaya, East Tien Shan, and Hengduan Shan under all scenarios. In the Hissar Alay, Pamir, and Hindu Kush regions, glacier peak water is expected to occur around 2050 under all SSP scenarios. For Karakoram, peak water is projected to occur around 2080 under SSP245 and SSP585; however, in the 21st century, glacier peak water may not occur in Karakoram under SSP370 or in West Kunlun under SSP370 and SSP585. It is projected to appear substantially later for Hissar Alay, Pamir, West Tien Shan, West Kunlun, East Kunlun, Inner Tibet, Hindu Kush, and Karakoram, with peak water not anticipated before the 2050s under SSP585. (2) For the entire HMA, glacier elevation and latitude are the most significant factors influencing peak water timing under all emission scenarios, with average correlation coefficients of 0.48 (*P* < 0.01) and 0.47 (*P* < 0.01), respectively. Elevation is the most relevant factor in Pamir, West Tien Shan, East Tien Shan, East Kunlun, Qilian Shan, Hindu Kush, Karakoram, and East Himalaya, while latitude is the most relevant factor in Inner Tibet. Larger glacier areas delay peak water timing across all subregions, particularly in West Kunlun and West Himalaya, where the mean glacier areas are 1.51 km² and 0.79 km², respectively. Peak water occurs earlier in glaciers with steeper slopes, which facilitate glacial movement and reduce glacier storage capacity. Debris cover also influences peak water timing: thinner debris cover (< 5 cm) accelerates energy absorption, leading to earlier peak water, while thicker debris cover insulates against radiation, resulting in later peak water.

## Data availability

The Randolph Glacier Inventory data is available at https://www.glims.org/RGI/randolph60.html (RGI v6.0) [8]. Supraglacial debris thickness data were obtained from the dataset generated by McCarthy [32]. Future glacier runoff data were collected from Global PyGEM-OGGM Glacier Projections Version 1 [28].

## Declaration of competing interest

The authors declare that they have no conflicts of interest in this work.

## Appendix Supplementary materials

Image, application 1
