# Supplementary material for: Variations in glacier peak water timing and its influencing factors in High-Mountain Asia
Source: Fundam Res. 2024 Dec 27;6(1):324–34. doi: 10.1016/j.fmre.2024.12.006 (PMC12869767; doi:10.1016/j.fmre.2024.12.006)
Supplement: Supplementary file 1 [file mmc1.docx]

**Variations in glacier peak water timing and its influencing factors in High-Mountain Asia**

**Haodong Lyu^1,2^, Gonghuan Fang^1,2*^, Yaning Chen^1,2*^, Wenting Liang^1,2^, Zewei Qiu^1,2^, Yupeng Li^1,2^, Weili Duan^1,2^ and Zhi Li^1,2^**

^1^State Key Laboratory of Desert and Oasis Ecology, Key Laboratory of Ecological Safety and Sustainable Development in Arid Lands, Xinjiang Institute of Ecology and Geography, Chinese Academy of Sciences, Urumqi, China

^2^University of Chinese Academy of Sciences, Beijing, China

Corresponding authors: Gonghuan Fang (fanggh@ms.xjb.ac.cn)

Yaning Chen ([chenyn@ms.xjb.ac.cn)](mailto:chenyn@ms.xjb.ac.cn))

1. **Global Climate Models (GCMs)**

We employed 12 Global Climate Models (GCMs) from CMIP6 to assess the peak water timing in the future, including BCC-CSM2-MR, CESM2, CESM2-WACCM, EC-Earth3, EC-Earth3-Veg, FGOALS-f3-L, GFDL-ESM4, INM-CM4-8, INM-CM5-0, MPI-ESM1-2-HR, MRI-ESM2-0, and NorESM2-MM. These models represent a diverse set of simulations used to understand the impacts of climate change across various scenarios. It has been suggested that these models perform well in simulating future atmospheric conditions in High Mountain Asia (HMA) [1, 2]. Additionally, NorESM2-LM has been identified as one of the most effective GCMs for this region [1].

**Table S1 Basic information of the 12 Global climate model (GCM)s sed in this study.**

| ID | Model name | Country | Institution | Atmospheric resolution  (lon × lat) |
| --- | --- | --- | --- | --- |
| A | BCC-CSM2-MR | China | BCC | 1.12° × 1.12° |
| B | CESM2 | USA | NCAR | 1.25° × 0.9° |
| C | CESM2-WACCM | USA | NCAR | 1.25° × 0.9° |
| D | EC-Earth3-Veg | Europe | EC-Earth-Cons | 0.7° × 0.7° |
| E | EC-Earth3 | Europe | EC-Earth-Cons | 0.7° × 0.7° |
| F | FGOALS-f3-L | Europe | CAS | 1.25° × 1° |
| G | GFDL-ESM4 | USA | NOAA-GFDL | 1.25° × 1° |
| H | INM-CM4–8 | Russia | INM | 2° × 1.5° |
| I | INM-CM5–0 | Russia | INM | 2° × 1.5° |
| J | MPI-ESM1–2-HR | Germany | DKRZ | 0.94° × 0.93° |
| K | MRI-ESM2–0 | Japan | MRI | 1.12° × 1.12° |
| L | NorESM2-MM | Norway | NCC | 1.25° × 0.94° |

1. **PyGEM-OGGM**

In this study, the future glacier runoff data were generated by the glacier model PyGEM-OGGM version 1 [3]. The model accounts for glacier mass balance (i.e., snow accumulation minus melt plus refreezing) for each surface elevation bin with energy balance models and temperature index models on a monthly time step. The glacier melt is computed using a degree-day model, accumulation using a temperature threshold, and refreezing based on the annual air temperature [4]. The monthly mass balance mi at elevation z is computed as follows:

$$\begin{aligned} m_{i}\left( z \right)=p_{f}P_{i}^{Solid}\left( z \right)-\mu^{*}\max\left( T_{i}\left( z \right)-T_{Melt}, 0 \right)+\varepsilon\#\left( 1 \right) \end{aligned}$$

where $P_{i}^{Solid}\left( z \right)$ is the monthly solid precipitation, $p_{f}$ is a global precipitation correction factor (defaults to 2.5), $\mu^{*}$ is the glacier's temperature sensitivity, $T_{i}(z)$is the monthly air temperature at altitude $z$. $T_{Melt}$is the monthly air temperature above which ice melt is assumed to occur, and ε is a residual (or bias correction) term. The parameter μ* indicates the glacier sensitivity to temperature which needs to be calibrated.

The OGGM model was forced by twelve General Circulation Models (GCMs) under the four Shared Socioeconomic Pathway (SSP) scenarios. These twelve GCMs were presented in Section “2.3 Future glacier runoff data”.

In the model, the DEM data was used as input in the OGGM model, which automatically derives the topographic factors (i.e., glacier slope, aspect, and elevation). These factors are crucial for calculating ice flow and determining the temperature and heat balance of each glacier. Elevation influences climate through its elevation-dependent effects on precipitation and temperature. Additionally, latitude affects atmospheric circulation. Glacier area was used to calculate the flux of ice $q$ (m3 s−1) through a glacier flux-gate (cross section) of area $S$ (m2) as follows:

$$\begin{aligned} q=uS\#\left( 2 \right) \end{aligned}$$

where $u$ is the average velocity (m s−1). To simulate the melt rate of debris-covered glaciers, spatially distributed sub-debris melt enhancement factors are used to account for the enhanced or suppressed melting due to debris thickness.

1. **References**

[1] R. Chen, K. Duan, W. Shang, P. Shi, Y. Meng and Z. Zhang, Increase in seasonal precipitation over the Tibetan Plateau in the 21st century projected using CMIP6 models, Atmos. Res. 277 (2022) 106306

[2] Y. Liu, J. Gao and Y. Wang, Evaluation of atmospheric moisture transport to the Tibetan Plateau from 33 CMIP6 models, npj Clim. Atmos. Sci. 7 (2024) 231

[3] D. R. Rounce, R. Hock, F. Maussion, R. Hugonnet, W. Kochtitzky, M. Huss, E. Berthier, D. Brinkerhoff, L. Compagno, L. Copland, D. Farinotti, B. Menounos and R. W. McNabb, Global glacier change in the 21st century: Every increase in temperature matters, Science 379 (2023) 78-83

[4] F. Maussion, A. Butenko, N. Champollion, M. Dusch, J. Eis, K. Fourteau, P. Gregor, A. H. Jarosch, J. Landmann, F. Oesterle, B. Recinos, T. Rothenpieler, A. Vlug, C. T. Wild and B. Marzeion, The Open Global Glacier Model (OGGM) v1.1, Geosci. Model Dev. 12 (2019) 909-931


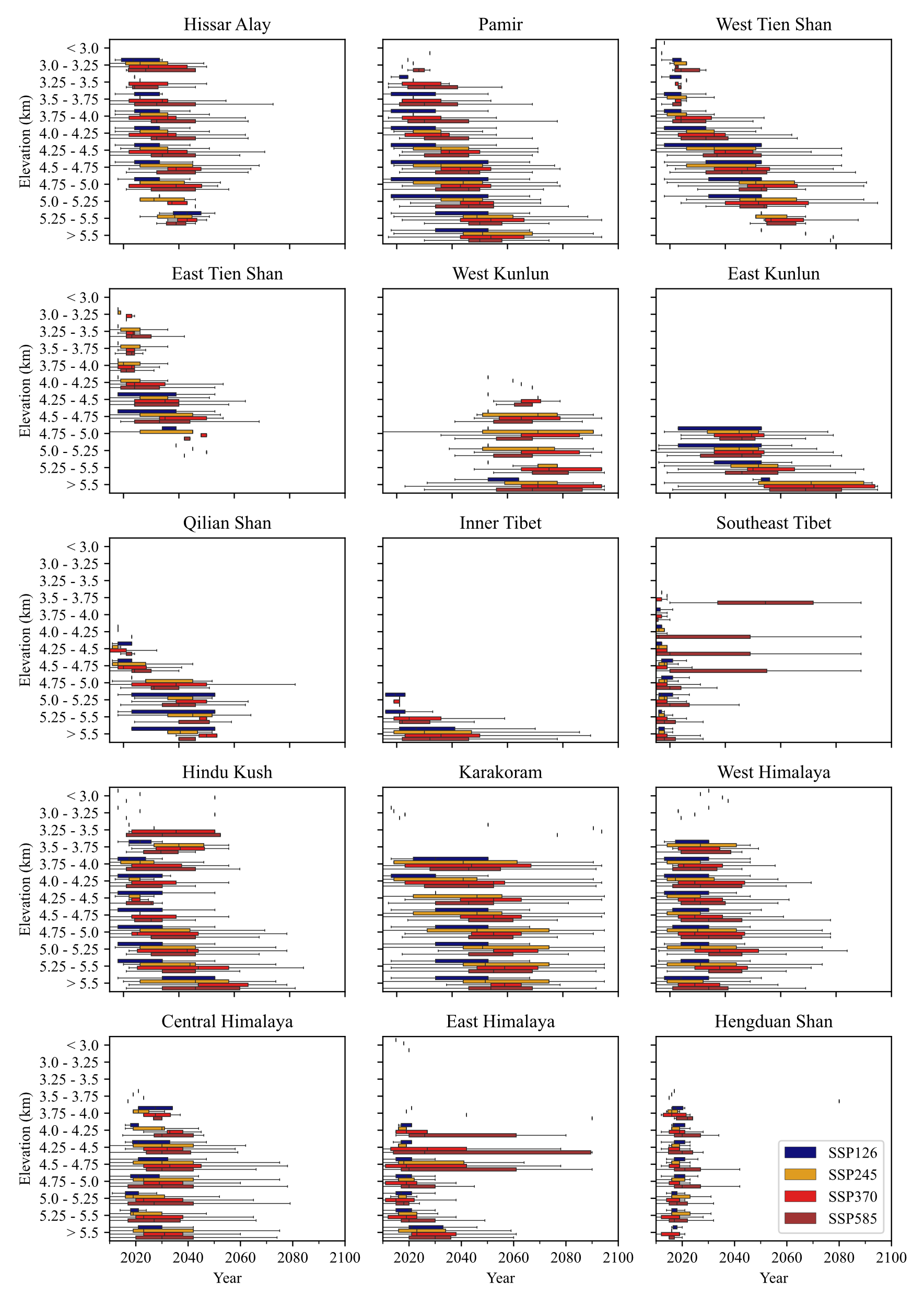


**Fig S1 Peak water timing distribution in multiples of elevation ranges in each subregion.** The glaciers are divided into 12 ranges by the glacier elevation and the colors represent the four SSPs. The box represents the interquartile range of the peak water timing, which contains the median 50% of the values. The bottom and top edges of the box indicate the first quartile and the third quartile, respectively. The earliest and latest peak water within the range of the whiskers are the minimum and maximum values, respectively.


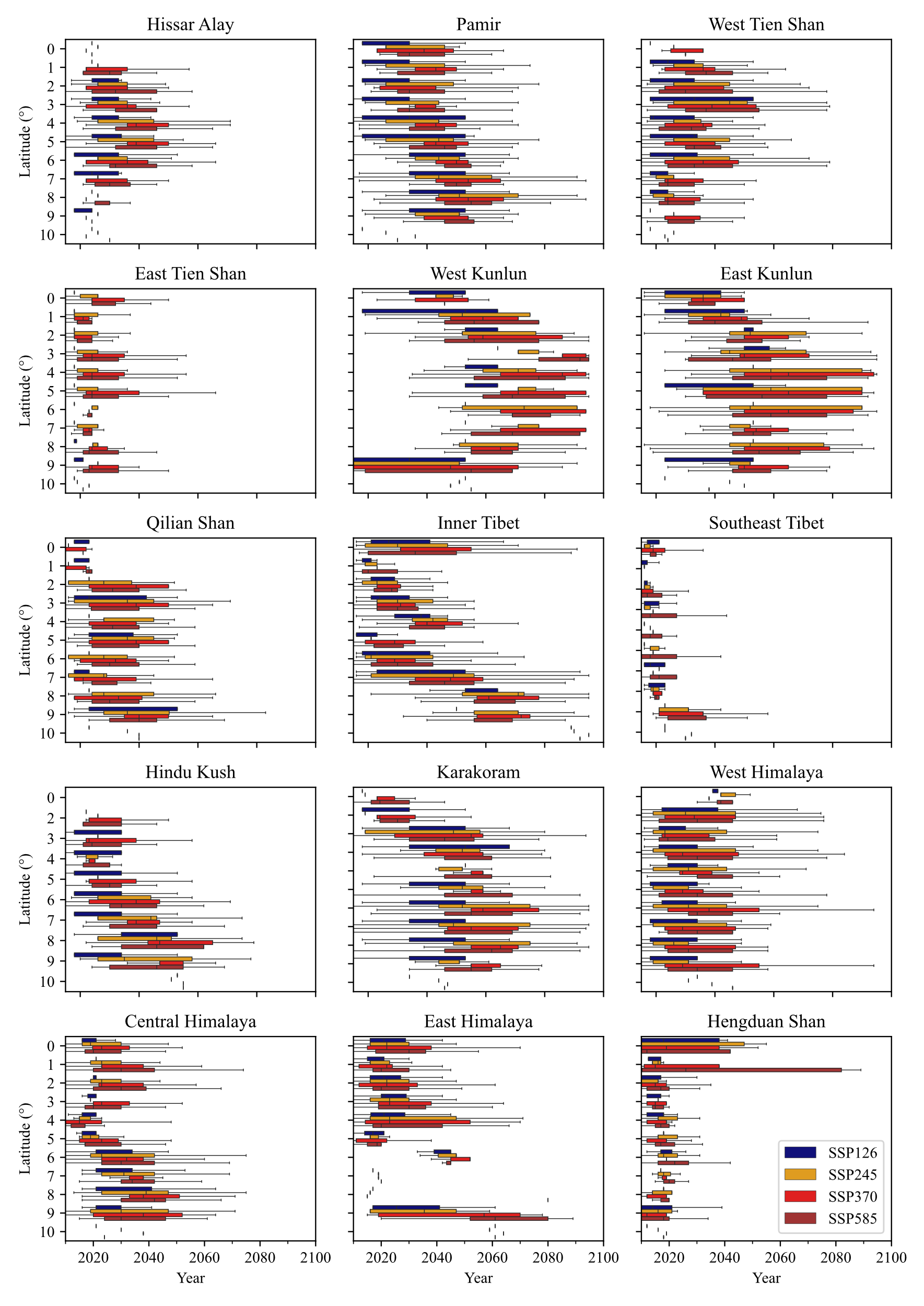


**Fig S2 Peak water timing distribution in multiples of latitude ranges.** The glaciers are divided into 10 equal ranges by the glaciers’ latitude. The box represents the interquartile range of the peak water timing, which contains the median 50% of the values. The bottom and top edges of the box indicate the first quartile and the third quartile, respectively. The earliest and latest peak water within the range of the whiskers are the minimum and maximum values, respectively.


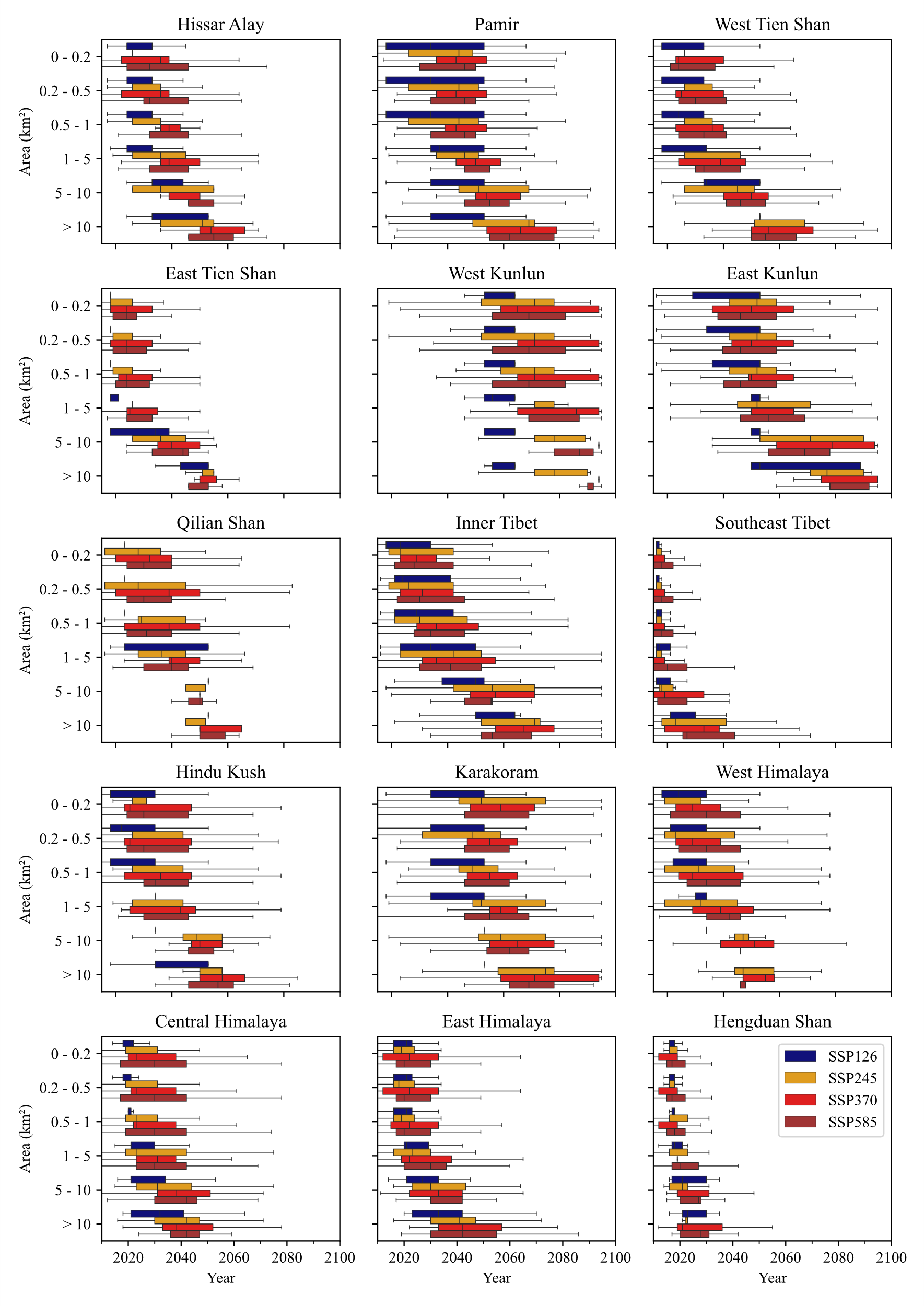


**Fig S3 Peak water timing distribution in multiples of area ranges.** The glaciers are divided into 6 ranges by the glacier area and the colors represent the four SSP scenarios.The box represents the interquartile range of the peak water timing, which contains the median 50% of the values. The bottom and top edges of the box indicate the first quartile and the third quartile, respectively. The earliest and latest peak water within the range of the whiskers are the minimum and maximum values, respectively.


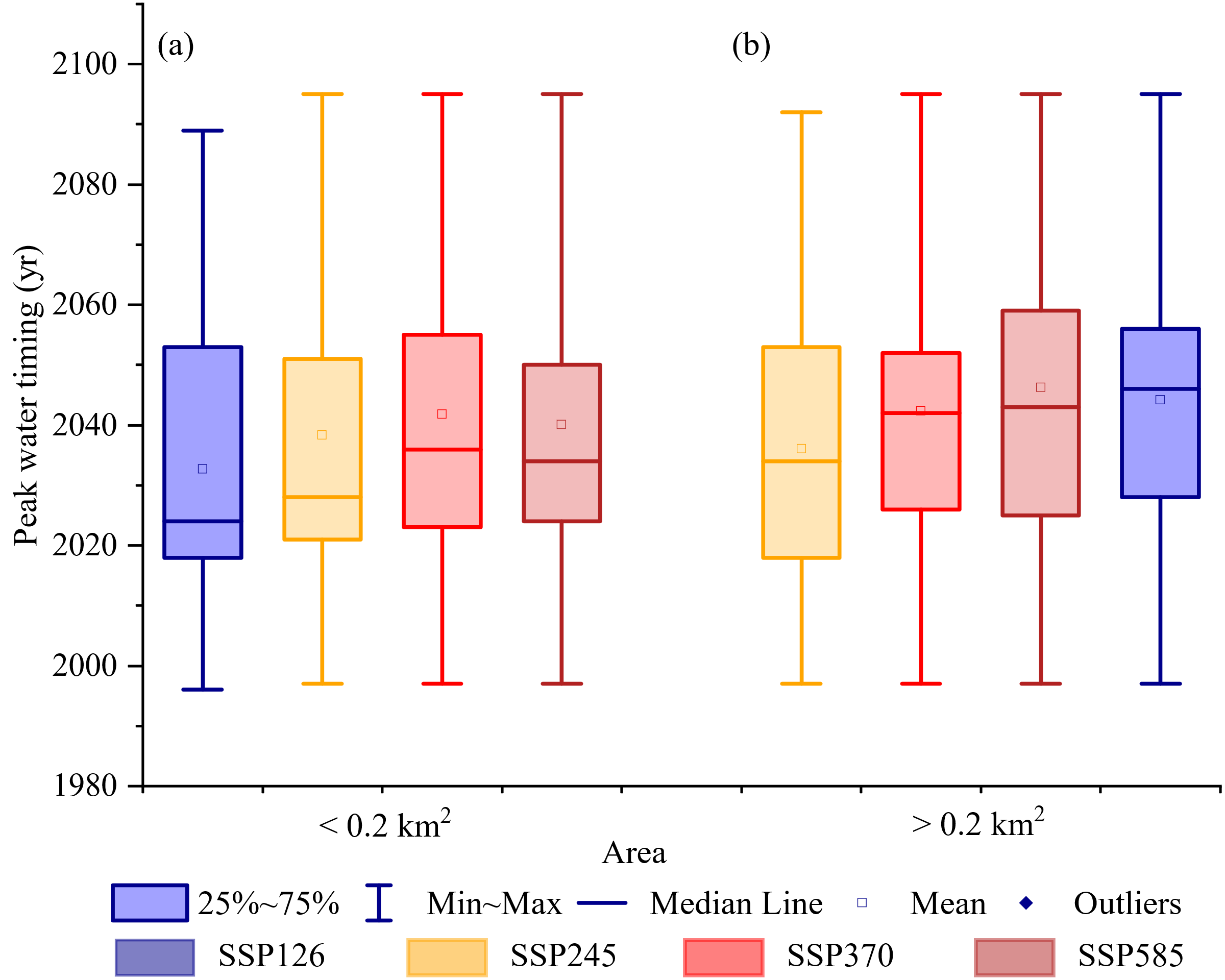


**Fig S4 Peak water timing distribution in the glacier (a) smaller and (b) larger than 2 km^2^.** The box represents the interquartile range of the peak water timing, which contains the median 50% of the values. The bottom and top edges of the box indicate the first quartile and the third quartile, respectively. The earliest and latest peak water within the range of the whiskers are the minimum and maximum values, respectively. The median line represents the median peak water timing in each ranges.


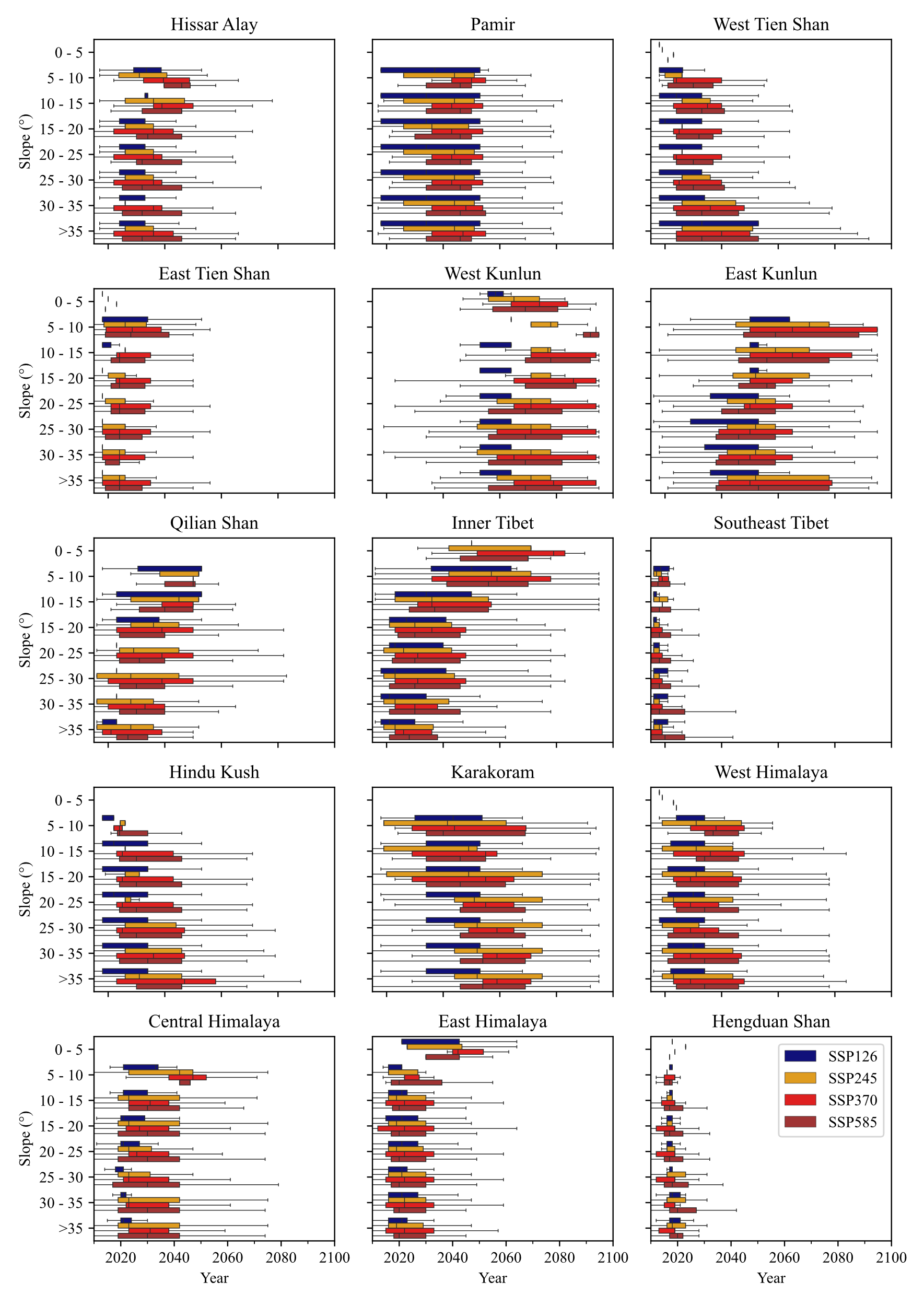


**Fig S5 Peak water timing distribution in multiples of slope ranges.** The glaciers are divided into 8 ranges by the glacier slope and the colors represent the four SSP scenarios.The box represents the interquartile range of the peak water timing, which contains the median 50% of the values. The bottom and top edges of the box indicate the first quartile and the third quartile, respectively. The earliest and latest peak water within the range of the whiskers are the minimum and maximum values, respectively.


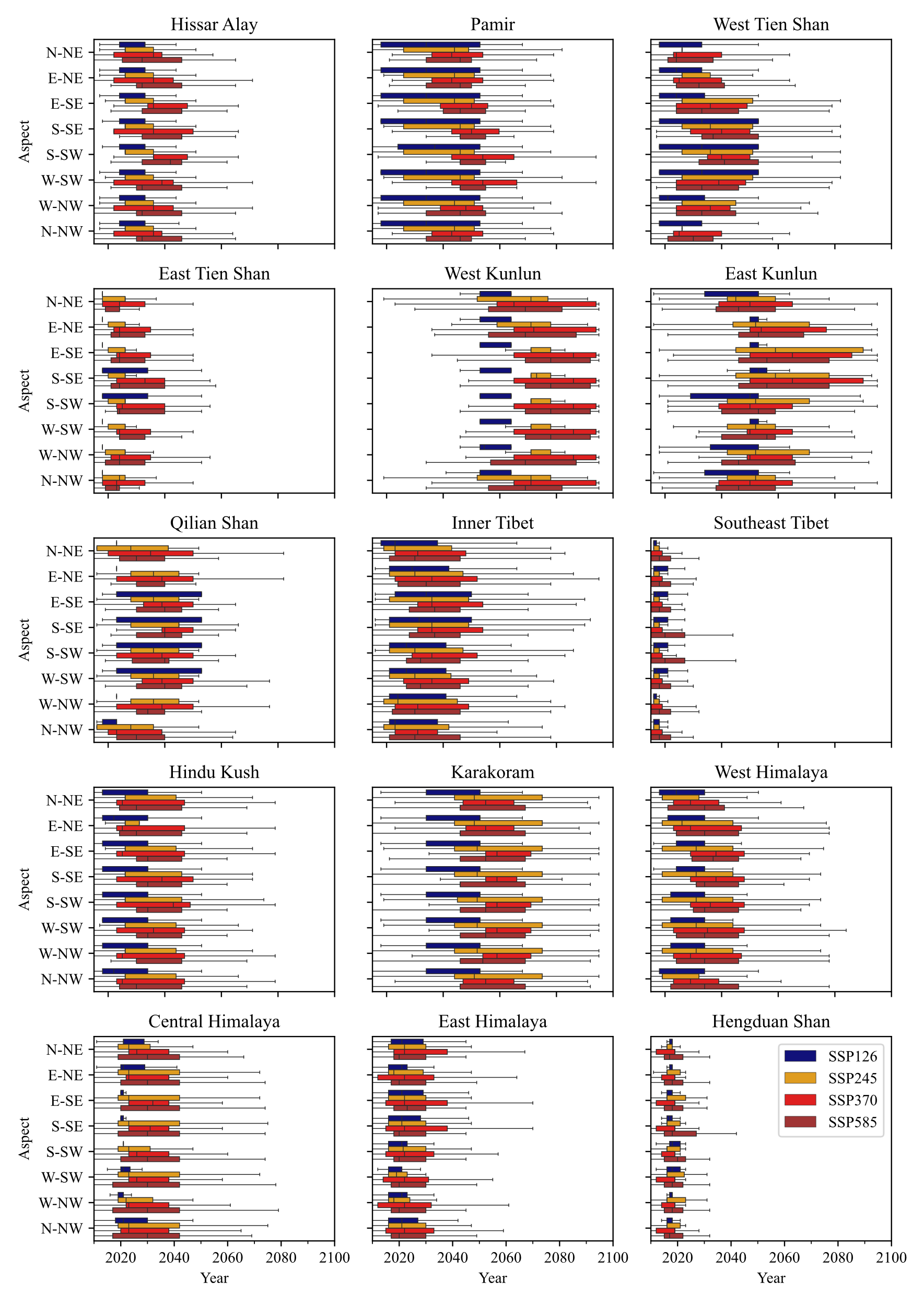


**Fig S6 Peak water timing distribution in each aspect.** In the plot, the aspect was divided into 8 aspects with N-NE, E-NE, E-SE, S-SW, W-SW, W-NW, and N-NW. And the colors represent the four SSP scenarios.The box represents the interquartile range of the peak water timing, which contains the median 50% of the values. The bottom and top edges of the box indicate the first quartile and the third quartile, respectively. The earliest and latest peak water within the range of the whiskers are the minimum and maximum values, respectively.


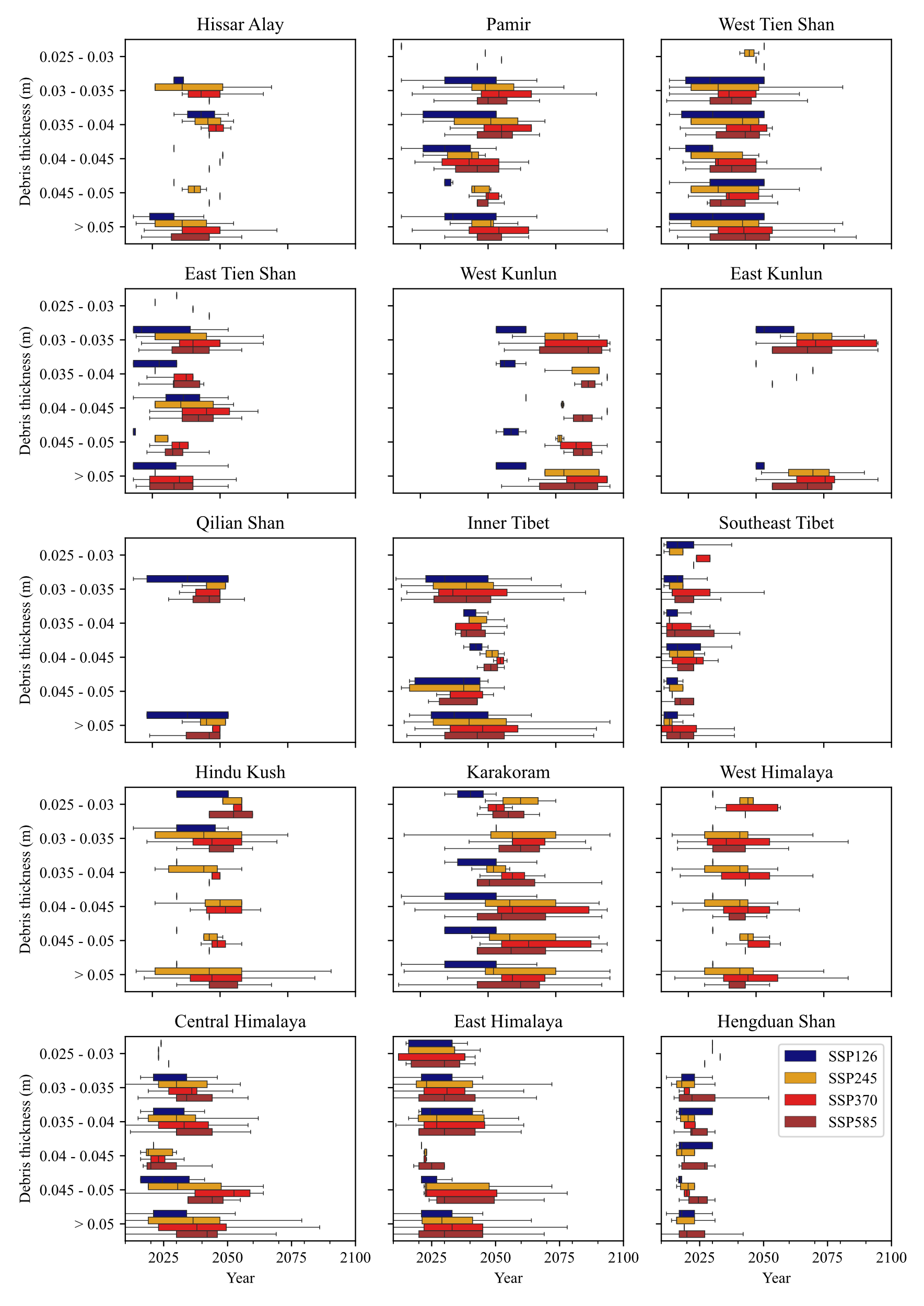


**Fig S7 Peak water timing distribution in multiples of subglacial debris thickness ranges.** The glaciers are divided into 6 ranges by the glacier debris thickness and the colors represent the four SSP scenarios.The box represents the interquartile range of the peak water timing, which contains the median 50% of the values. The bottom and top edges of the box indicate the first quartile and the third quartile, respectively. The earliest and latest peak water within the range of the whiskers are the minimum and maximum values, respectively.


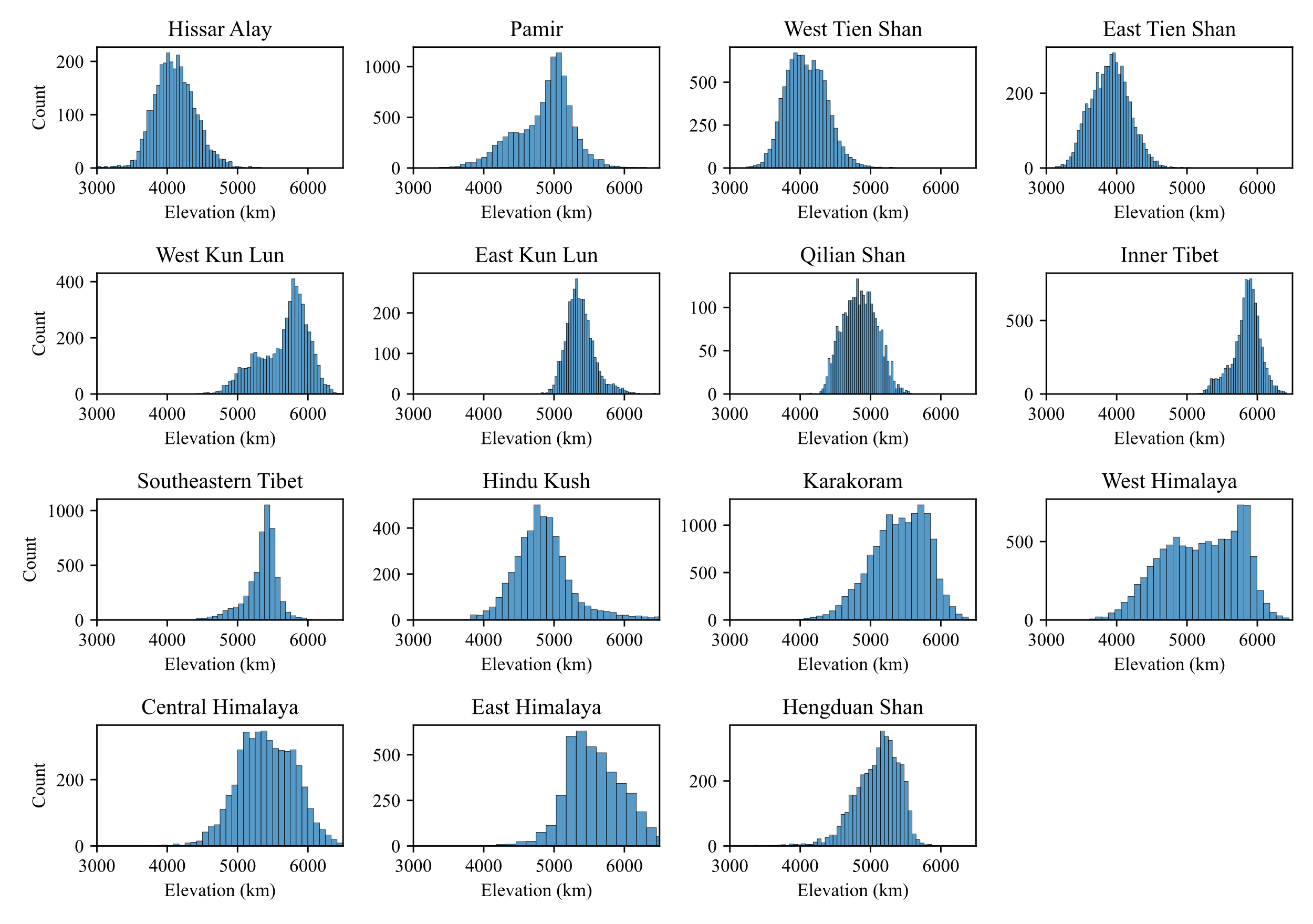


**Fig S8 Distribution of glaciers’ medium elevation in each subregion of HMA.** The histograms prohibit the distribution of count of glaciers in each range of elevation in each subregion. The range of elevation is 100m.


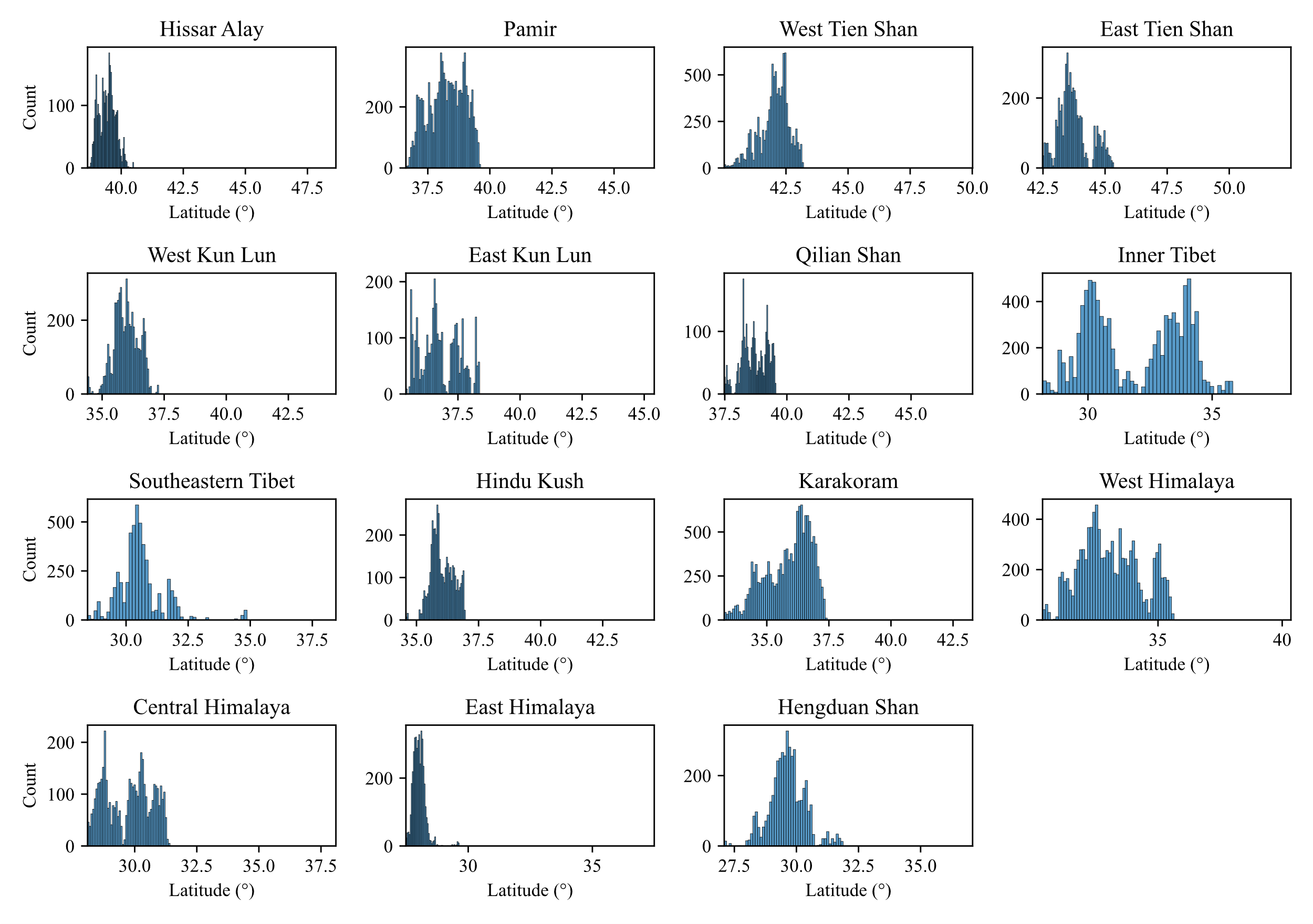


**Fig S9 Distribution of glaciers’ latitude in each region of HMA.** The histograms prohibit the distribution of count of glaciers in each range of latitude in each subregion.


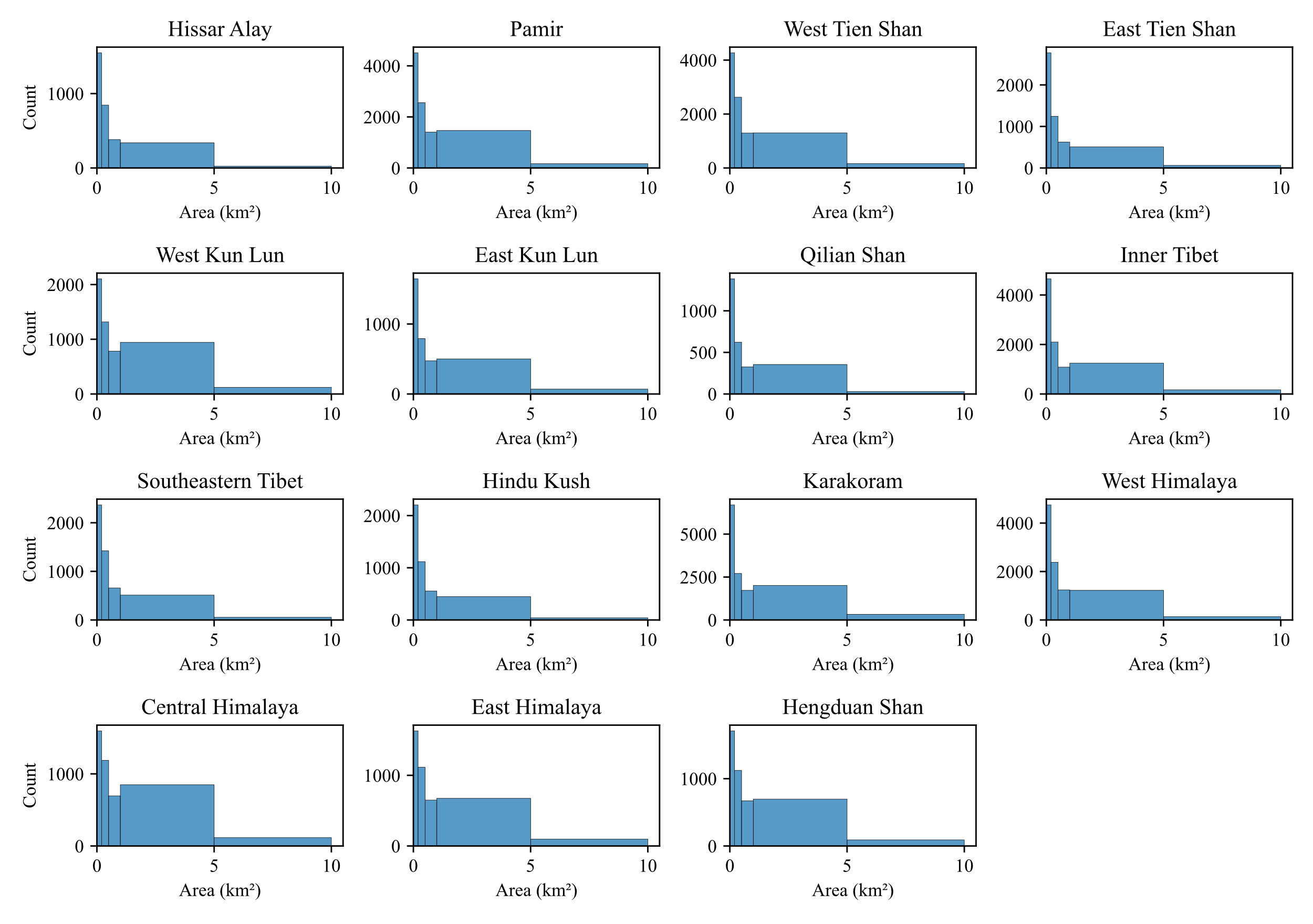


**Fig S10 Distribution of glaciers’ area in each region of HMA.** The histograms prohibit the distribution of count of glaciers in each range of area in each subregion. The area is divided into 5 parts with the intervals of 0.0.2km^2^, 0.5 km^2^, 1 km^2^, and 5 km^2^.


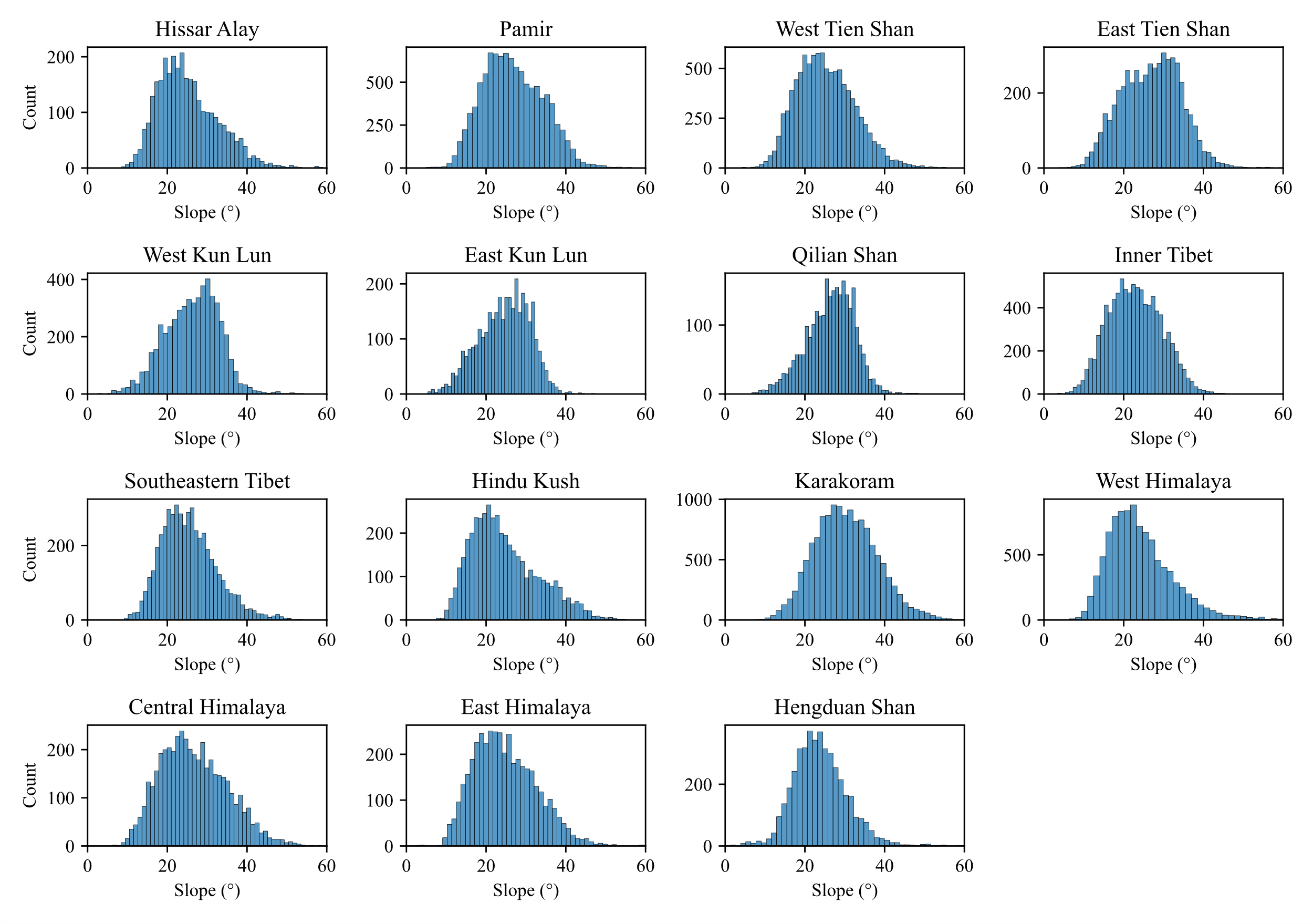


**Fig S11 Distribution of glaciers’ slope in each region of HMA.** The histograms prohibit the distribution of count of glaciers in each degree of slope in each subregion from 0 to 60°.


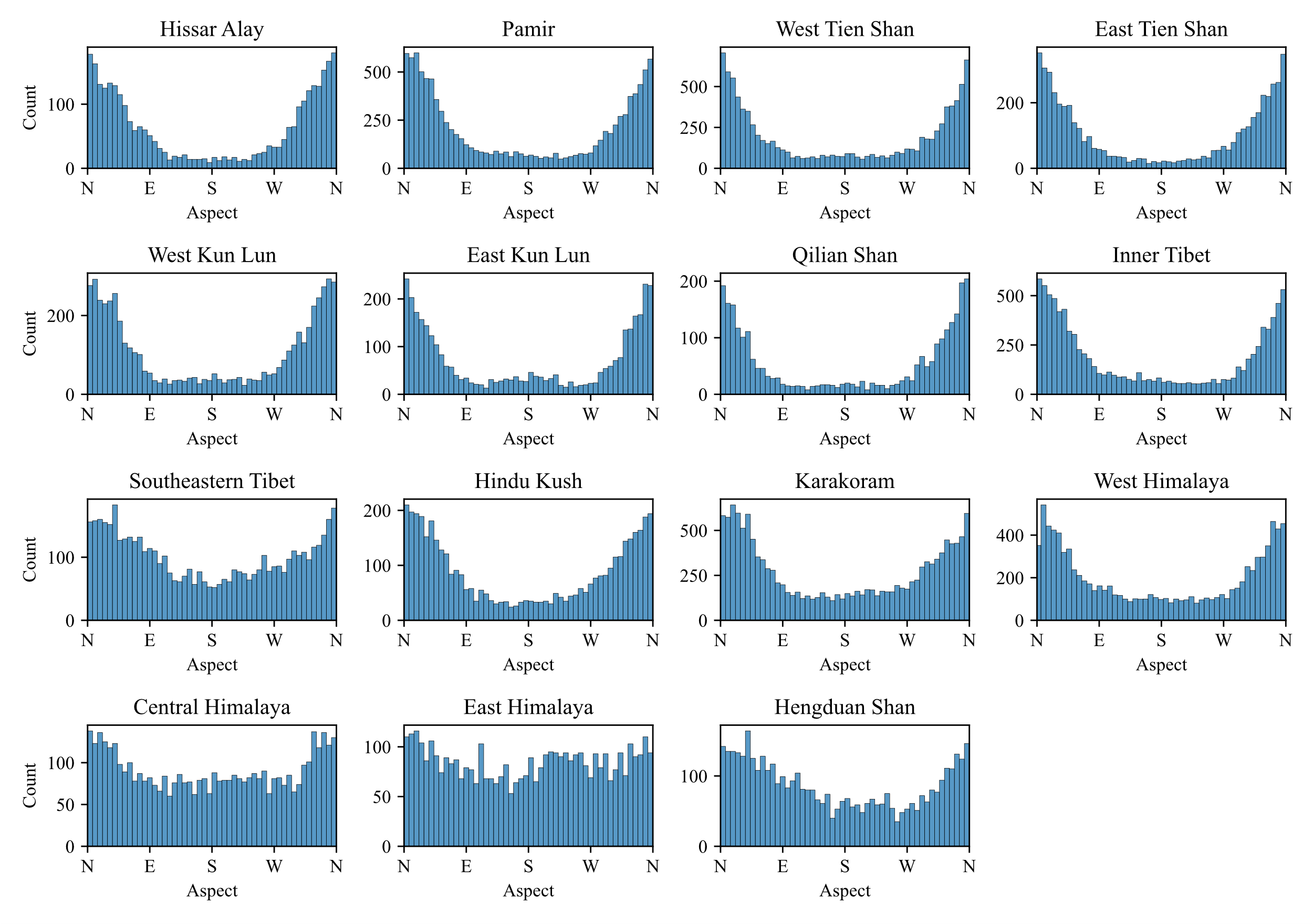


**Fig S12 Distribution of glaciers’ aspect in each region of HMA.** The histograms prohibit the distribution of count of glaciers in each range of aspect in each subregion. The aspect goes from north to south and then back to north.


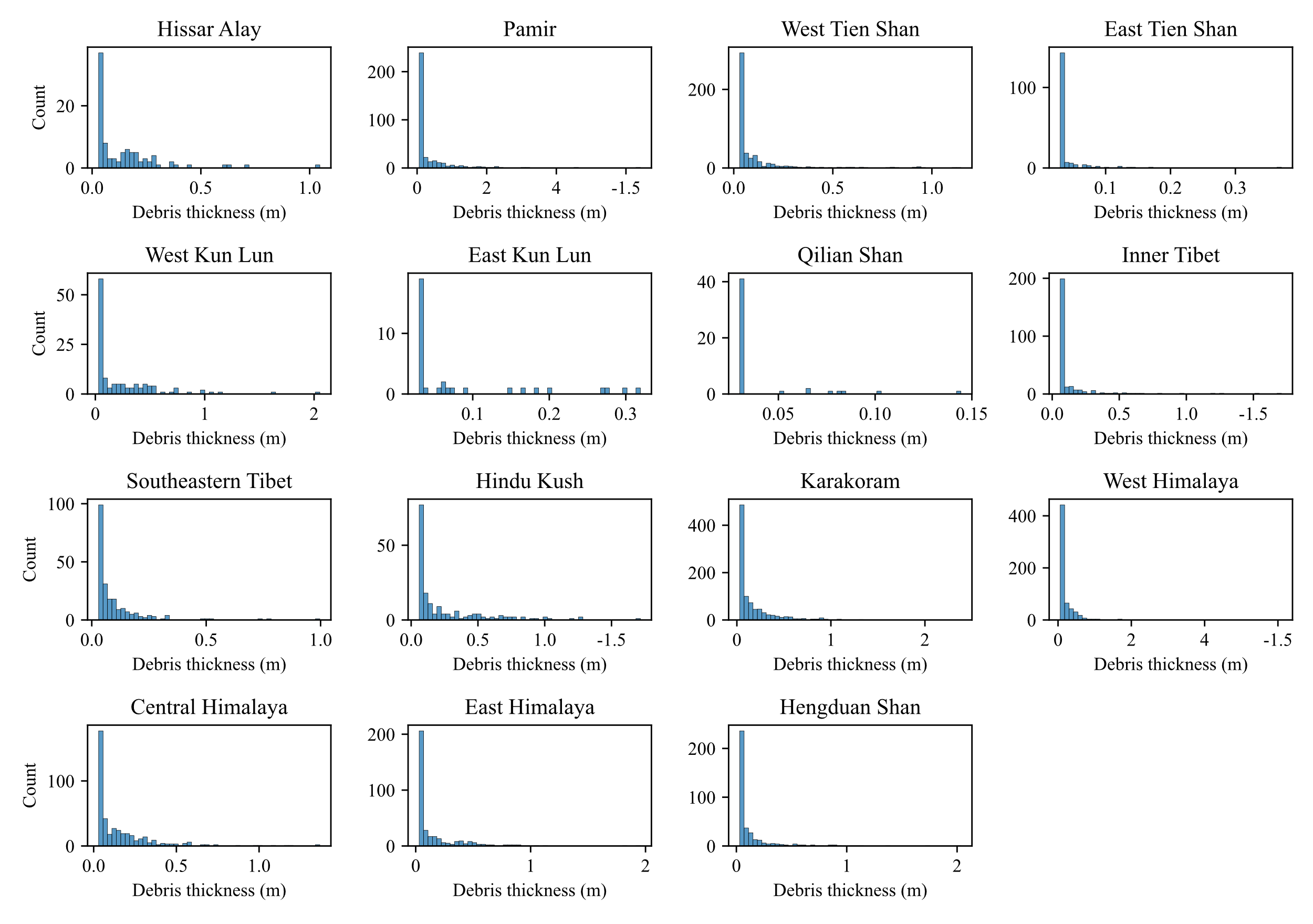


**Fig S13 Distribution of subglacial debris thickness in each region of HMA.** The histograms prohibit the distribution of count of glaciers in each range of debris thickness in each subregion.


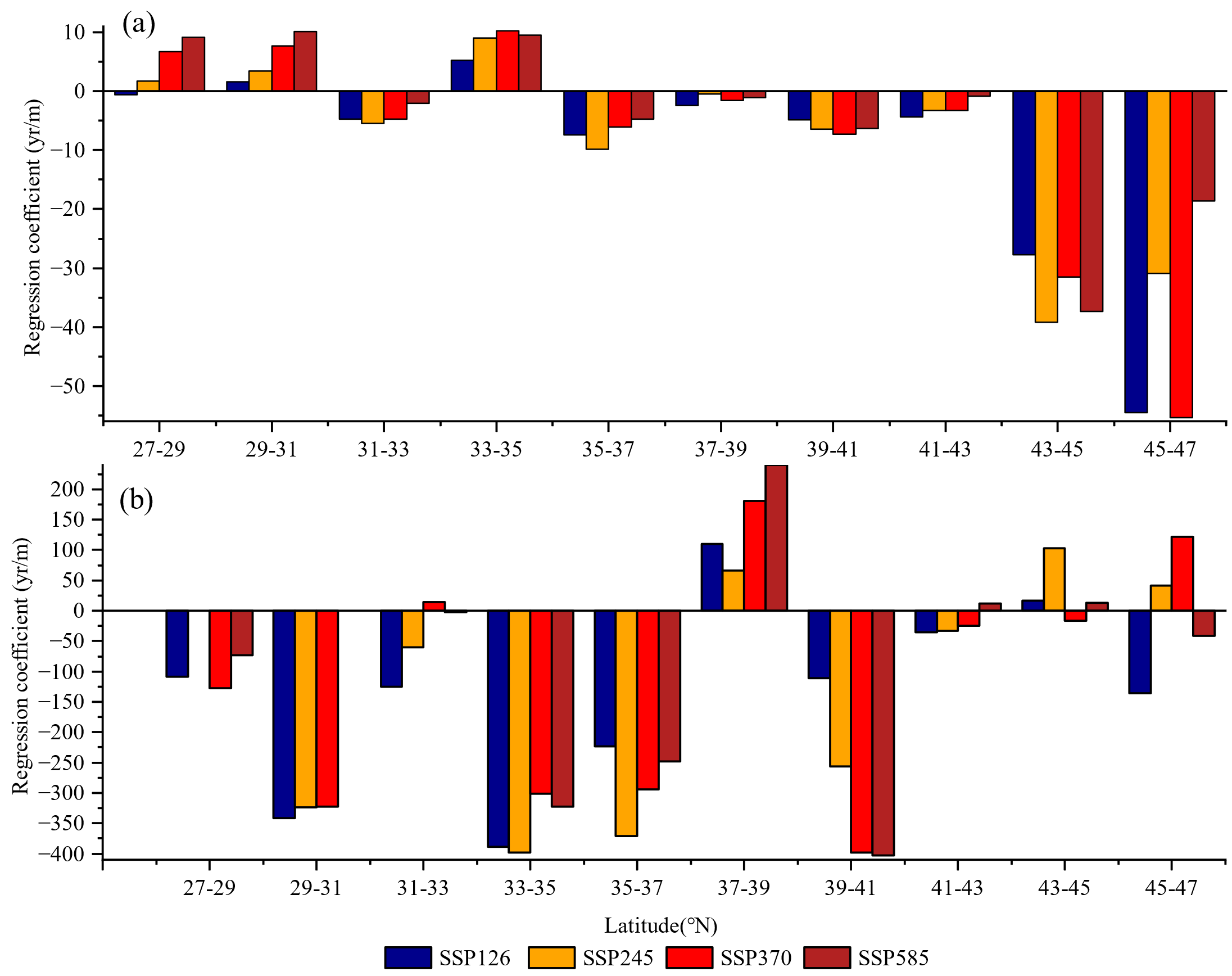


**Fig S14 The coefficient of the linear regression between peak water timing and debris thickness in each bins of latitude of (a) all the glaciers and (b) glaciers with debris thinner than 5cm.** In subfigure (b), the linear regression was conducted only within the glaciers where the debris is thinner than 5cm.

**

**

**Fig S15. Estimate value of result of Structural equation model of peak water timing and glacier attributes in each subregion.** The statistics for glacier attributes in each subregion are presented in Fig. 1.
